# A novel method for real-time analysis of the complement C3b:FH:FI complex reveals dominant negative *CFI* variants in age-related macular degeneration

**DOI:** 10.3389/fimmu.2022.1028760

**Published:** 2022-12-28

**Authors:** Thomas M. Hallam, Thomas E. Cox, Kate Smith-Jackson, Vicky Brocklebank, April J. Baral, Nikolaos Tzoumas, David H. Steel, Edwin K. S. Wong, Victoria G. Shuttleworth, Andrew J. Lotery, Claire L. Harris, Kevin J. Marchbank, David Kavanagh

**Affiliations:** ^1^ Translational and Clinical Research Institute, Newcastle University, Newcastle upon Tyne, United Kingdom; ^2^ National Renal Complement Therapeutics Centre, Royal Victoria Infirmary, Newcastle upon Tyne, United Kingdom; ^3^ Sunderland Eye Infirmary, Sunderland, United Kingdom; ^4^ Biosciences Institute, Newcastle University, International Centre for Life, Newcastle upon Tyne, United Kingdom; ^5^ Clinical and Experimental Sciences, Faculty of Medicine, Southampton General Hospital, University of Southampton, Southampton, United Kingdom; ^6^ National Institute for Health and Care Research (NIHR) Newcastle Biomedical Research Centre, Biomedical Research Building, Newcastle upon Tyne, United Kingdom

**Keywords:** AMD (age-related macular degeneration), aHUS (atypical haemolytic uraemic syndrome), Factor I, Factor H, Complement, Surface plasmon resonance, C3G (C3 Glomerulopathy), PNH (paroxysmal nocturnal haemoglobinuria)

## Abstract

Age-related macular degeneration (AMD) is linked to 2 main disparate genetic pathways: a chromosome 10 risk locus and the alternative pathway (AP) of complement. Rare genetic variants in complement factor H (*CFH;* FH*)* and factor I (*CFI;* FI*)* are associated with AMD. FH acts as a soluble cofactor to facilitate FI’s cleavage and inactivation of the central molecule of the AP, C3b. For personalised treatment, sensitive assays are required to define the functional significance of individual AP genetic variants. Generation of recombinant FI for functional analysis has thus far been constrained by incomplete processing resulting in a preparation of active and inactive protein. Using an internal ribosomal entry site (IRES)-Furin-*CFI* expression vector, fully processed FI was generated with activity equivalent to serum purified FI. By generating FI with an inactivated serine protease domain (S525A FI), a real-time surface plasmon resonance assay of C3b:FH:FI complex formation for characterising variants in *CFH* and *CFI* was developed and correlated well with standard assays. Using these methods, we further demonstrate that patient-associated rare genetic variants lacking enzymatic activity (e.g. *CFI* I340T) may competitively inhibit the wild-type FI protein. The dominant negative effect identified in inactive factor I variants could impact on the pharmacological replacement of FI currently being investigated for the treatment of dry AMD.

## Introduction

Age -related macular degeneration (AMD) is the most common cause of irreversible vision loss in developed nations. The global prevalence of AMD is ~8.3% in those over 65 and it is projected to afflict 288 million by 2040 (1), representing a substantial global health and economic burden. There are two subtypes of AMD: wet and dry. Wet AMD involves angiogenesis in the choroid and/or macular neovascularisation which can rapidly progress to a disciform macular scar and legal blindness. Inhibition of vascular endothelial growth factor with monoclonal antibodies can preserve vision but many patients with wet AMD ultimately lose vision due to progressive dry AMD. Dry AMD is driven by degeneration or geographic atrophy (GA) of the retinal pigment epithelium. There is currently no treatment for GA.

In addition to behavioural and environmental factors, >30 common genetic loci have been confirmed to be associated with AMD (2). The 2 main loci identified involve: the activation of the complement system (3–9); and the age-related maculopathy susceptibility 2/high-temperature requirement A serine peptidase 1 *(ARMS2-HTRA1)* risk locus linked to overexpression of HTRA1 and accumulation of macrophages in the subretinal space with resultant inflammation (10).

The complement system is part of the innate immune response to pathogens, and it is strictly regulated to prevent collateral damage to self-tissues (11). The alternative pathway (AP) of complement is a positive amplification loop accounting for ~80-90% of all terminal pathway activation (12). It is the components [C3 (4), factor B (FB) (5)] and regulators [Factor I (FI) (3), Factor H (FH)] (6–9) of the AP that are most strongly linked to AMD. C3 is the central component of the AP and interacts with FB and Factor D (FD) to form the AP C3 convertase, C3bBb, which cleaves more C3 and results in the generation of downstream effector molecules e.g. C5a and the membrane attack complex. Regulation of the AP convertase is brought about by cleavage of C3b by the enzyme FI in association with one of its cofactors (e.g. FH) after the formation of an AP regulatory trimolecular complex (TMC) (e.g. C3b:FH:FI).

Common genetic variants in *C3*, *CFH* and *CFI* were initially associated with AMD in single nucleotide polymorphism association studies (3, 4, 6–9), however, more recently next generation sequencing studies have demonstrated an increased burden of rare genetic variants in *C3*, *CFH* and *CFI* in AMD (4, 13–18). Most of the disease-associated variants are individually rare, and a significant proportion of variants consist of missense mutations of unknown significance.

Clinical trials of FH and FI supplementation (Gemini’s GEM103; ClinicalTrials.gov Identifier: NCT04684394, and Gyroscope’s GT005; ClinicalTrials.gov Identifier: NCT04437368) respectively, have begun in AMD patients. While quantitative genetic defects in FI and FH are relatively straightforward to detect, and those in FI have been strongly associated with AMD, >50% of the rare genetic variants in *CFI* and *CFH* result in a protein that is secreted with unknown functional activity. With divergent pathways resulting in GA, defining the significance of rare genetic variants is critical to tailor appropriate pharmacological intervention.

Generation of recombinant FI has until now resulted in a mixture of inactive pro-I and fully processed FI. Full processing of FI requires cleavage by Furin of an RRKR linker at Arg339 followed by removal of the linker amino acids by carboxypetidases (19). So far, studies analysing FI are limited by this and require either co-transfection or downstream processing with Furin to generate a pure product. In addition, functional assays rely mainly on fluid phase C3b cleavage assays with various cofactors (20–23), although more complex zebrafish assays have also been utilised (24). These methods, however, do not enable real-time visualisation of the formation of C3b:cofactor:FI complexes.

**Figure 1:**
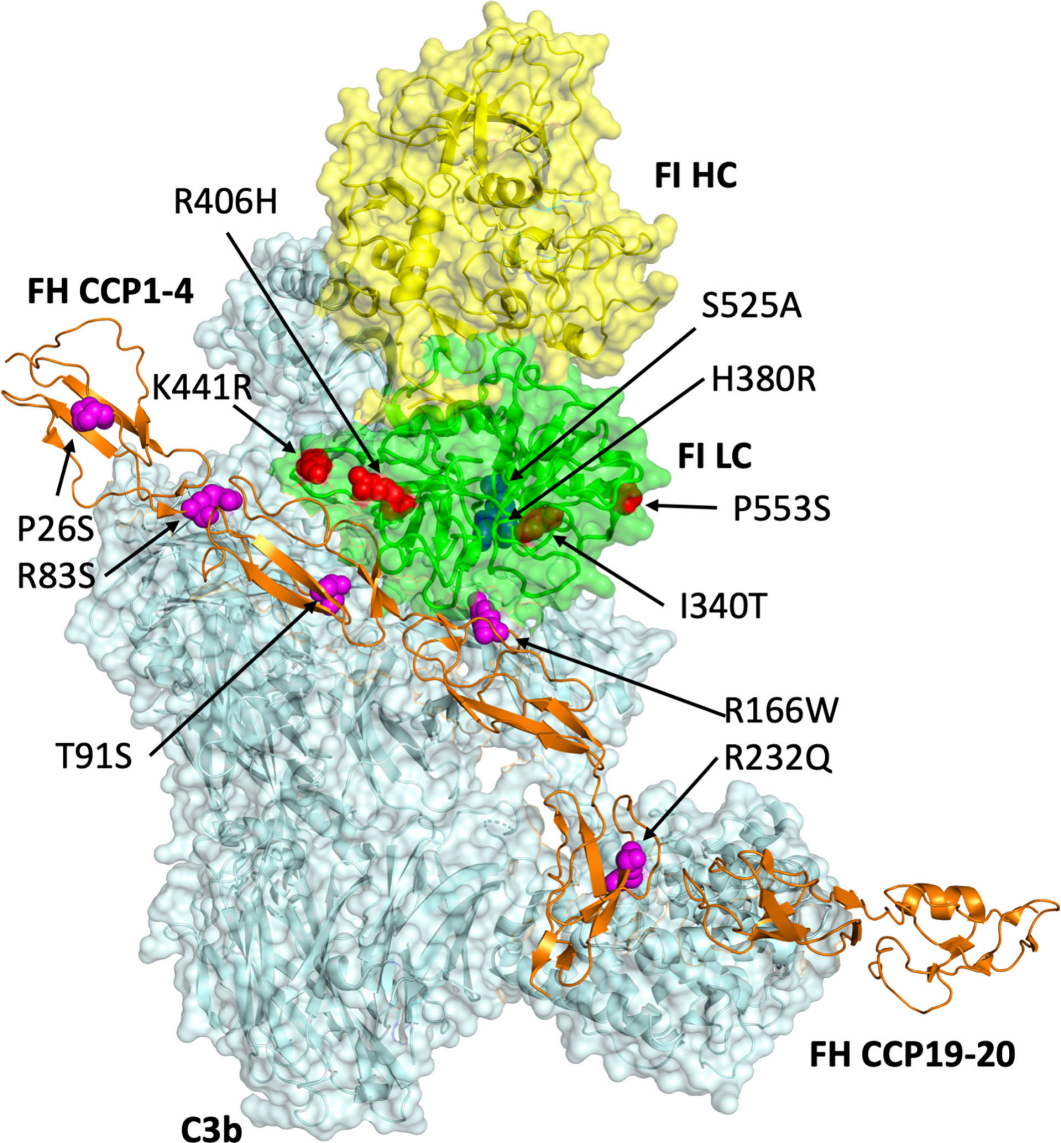
Modelling of genetic variants in *CFI* and *CFH*. Produced in Pymol V2.0 (Schrodinger LLC) using the PDB file 5o32 (Xue et al., 2017), this 3D model of FI (heavy chain: yellow; light chain: green) bound to C3b (pale cyan) and FH CCPs 1-4_19-20 (orange) displays the putative sites of amino acid residues altered by the mutations (FI: red spheres; FH: magenta spheres) identified in *CFI* and *CFH* interrogated by the present study. The FI catalytic triad including S525 is shown by blue spheres.

To overcome the aforementioned difficulties, a mechanism for the generation of fully processed recombinant FI was developed. The recombinantly produced FI had equivalent activity to serum-purified wild type protein. A mutation in FI previously utilised for crystallography of the AP regulatory (25) was generated to abrogate the cleavage of C3b and probe the real-time formation of the AP regulatory TMC. The utility of the inactive variant was demonstrated through analysis of a series of rare genetic variants in *CFH* and *CFI* reported in disease (**Figure 1**). Furthermore, we demonstrate that the AMD-linked FI variant I340T displays a competitive inhibitory effect on WT regulatory function which has implications for pharmacological supplementation strategy.

## Methods

### Protein production

Generation of FH CCP 1-4 variants in *Pichia Pastoris* (KM71H, ThermoFisher) was performed as previously described (26, 27). Primers for *CFH*1-4 mutagenesis are described in **Supplemental Table 1**.

FI variants were produced in HEK293T (ThermoFisher) using a polycistronic IRES vector with *Furin* and the full *CFI* cDNA sequence (NM_000204.4) designed using VectorBuilder. Full details are described at en.vectorbuilder.com/vector/VB171219-1127wqz.html. Site-directed mutagenesis was performed as described previously to introduce single (active backbone) and double (inactive backbone; given variant plus S525A) within *CFI* before transfection of HEK293T with cDNA was performed using JetPEI (Polyplus). Primers for *CFI* mutagenesis are described in **Supplemental Table 2**. Single clones expressing FI were selected and standard culturing of HEK in multilayer flasks resulted in the generation of supernatant for purification of FI using NHS activated HiTrap (Cytivia) affinity columns coupled with OX21 (mAb to human FI, Merck, UK) and elution with 0.1M glycine pH 2.7.

### Fluid phase cofactor assays

#### Factor H CCP 1-4

To detect differences in efficacy of the various FH CCP 1-4 variants in acting as a fluid phase cofactor for FI in the AP regulatory TMC, each was tested in a standard C3b co-factor assay modified from Kavanagh et al. (20). In brief, 5μL of each FH1-4 protein [or full-length factor H (FLFH) as an additional control (Complement Technologies Inc. (CompTech), A137)], at 50ng/μL, was added to 1μL of C3b (A114, CompTech, 1μg/μL), and 5μL of 20ng/μL FI (A138, CompTech), made up to a total of 15μL with PBS and incubated for 30 mins at 37°C. The products were separated using a 10-20% SDS-PAGE gel under standard reducing conditions and visualised by Coomassie blue staining. Molecular weights were compared to a PageRuler™ prestained Protein ladder (10 – 250 kDa) (ThermoFisher). Breakdown of the C3b α’-chain for each test reaction was measured using densitometry performed in ImagStudio™ Lite (Licor) and normalised to the beta chain as a loading control. To control for inter-assay differences between 3 repeated measurements the normalised α’ chain -remaining result was given as a ratio of the normalised C3b α’ chain remaining of a negative control that was included in each assay (i.e. C3b and FI only).

#### Factor I

For each FI variant tested, purified FI was added to 1µL of C3b (CompTech, A114, 1µg/µL), and 2.5µL of 155ng/µL FLFH (CompTech, A137) or 200ng/µL FH1-4 (in-house, Pichia recombinant) in a 0.6mL Eppendorf. Each reaction mixture was made up to a total volume of 15µL with PBS and incubated at 37°C. The amount of FI and the incubation duration depended on the cofactor used. Thirty nanograms of FI and a 15-minute incubation was utilised for reactions with FH1-4, whereas FLFH reactions required 10ng FI and a 15-minute incubation. Density of the C3b α’ -chain remaining was measured and normalised as described for FH1-4 variant cofactor assays.

#### Dominant negative assay

Increasing concentrations of inactive FI variants were added to WT FI in standard fluid phase CA assay tests. Twenty μg/mL S525A FI, or 40µg/mL I340T or H380R FI was serial diluted resulting in 7.5μL of (40), 20, 10, 5, 2.5 (and 1.25) μg/mL S525A (or I340T or H380R FI) being added to each standard WT test (10µg/mL), respectively, before being incubated for 60 mins at 37°C and separated by SDS-PAGE. Normalised density of the α’ -chain remaining was calculated as described for FH1-4 cofactor assays.

### BIAcore SPR

#### C3b coupling amine and thiol

Using the BIAcore S200, a Series S carboxymethyl 5 (CM5) sensor chip (both Cytivia, UK) was coupled with either 800 or 1000 RU (response units) of C3b by standard amine coupling following manufacturer’s instructions (Amine Coupling Kit, Cytiva, UK). Briefly, purified C3b (CompTech, A114) was immobilised on a single flow cell of the CM5 chip by flowing 5μg/mL protein, diluted in 50mM sodium acetate at pH 4.5, whilst a second flow cell was activated and blocked for reference.

Alternatively, in order to couple C3b to the chip surface *via* nucleophilic attack on its thioester domain, after the immobilisation of a nidus of C3b by amine coupling as described previously (28) (100RU), FB and FD (CompTech, A135 and A136 at 500nM and 60nM, respectively) were injected for 60s at 10μL/min to build the AP C3 convertase on the chip-bound C3b (C3bBb). Next, C3 (A114, CompTech, at 0.1mg/mL) was injected immediately across the surface of the flow cell for 180s at 10μL/min, so that the C3 was cleaved to C3b by the chip-bound convertase. Rapid nucleophilic attack on the internal thioester resulted in covalent binding of C3b to the surface through an ester bond. Several subsequent cycles of convertase formation and C3 cleavage resulted in 800-1000RU of C3b covalently immobilised on the chip surface in a physiological orientation.

#### FH CCP 1-4 affinity to C3b

After size exclusion of the 5 FH1-4 variants and WT into HEPES buffered saline with surfactant Tween 20 (HBST) (10mM HEPES, 140mM NaCl, 0.05% surfactant P20) or PBS, the proteins were concentrated 10-fold using 10kDa molecular weight cut-off Vivaspin columns (Sigma-Aldrich). The 6 proteins (WT, P26S, R83S, T91S, R166W, R232Q) were flowed over both the blank flow cell (Fc) and the C3b -immobilised Fc (i.e., Fc1 to 2) at concentrations ranging from 20μM to 0.3125μM, produced by a serial 1:2 dilution in HBST or PBST (PBS with 0.05% Tween 20), for 90s at a rate of 30μL/min. Following an 120s dissociation period, the Fcs were regenerated between each variant injection using 10mM sodium acetate and 1mM NaCl for 45 seconds at 20μL/min. Data were collected at 40Hz with a 120s stabilisation period between each sample. Buffer-only controls were included at several points during the automated run and data were double-referenced to the blank Fc and blank injections. Steady state responses were plotted using the S200 BIAevaluation software (Cytiva) and utilised to calculate estimated affinity (K_D_) of the variants to C3b (in μM) for each variant.

#### DAA Assay for FH1-4 variants

FB and FD (CompTech, A135 and A136 at 500nM and 60nM, respectively) were injected for 90s onto an 800RU C3b amine coupled CM5 chip to build a surface bound C3 convertase. The C3bBb complex was allowed to decay for 90s before 250nM FH1-4 variants were injected for 60s and decay accelerating activity of the FH1-4 proteins were compared to a negative (no FH) control. Injections were made at 20µL/min and data was collected at 40Hz. HBST supplemented with 1 mM MgCl was used as the running and dilution buffer. The flow cells were regenerated after each convertase build with an injection of pH 4 10mM sodium acetate and 1M NaCl for 40 seconds at 30μL/min.

#### TMC building for FH CCP 1-4 and FI variants

For FH variant testing, inactive FI (S525A) at a constant (118nM) concentration was injected with FH1-4 serially diluted 1:2 from 118nM to 14.75nM in PBST+Mg^2+^ (PBS with 0.05% Tween 20 and 1mM MgCl_2_). Injections of FH1-4 (118-14.75nM) and FI (118nM) were made onto a C3b -coupled CM5 chip (1000RU thiol-coupled) at the given concentrations for 2 mins at 30 μl/min, with a 500 second dissociation time. These injections were all performed in PBST+Mg^2+^ buffer at 25°C. The flow cells were regenerated between each step with an injection of pH 4 10mM sodium acetate and 1M NaCl for 40 seconds at 30μL/min after the end of each test, prior to the next injection of TMC building component -containing buffer. In all experiments, FH1-4 and FI were injected independently to control for bimolecular complex responses. Data were collected at 40Hz.

For FI variant testing using the AP regulatory TMC building assay, FI variants on the inactive backbone were serially diluted 1:2 in PBST+Mg^2+^ buffer from 125nM to 15.625nM, whilst FH1- 4 was kept at a constant concentration of 125nM for each test injection and TMC build.

To test AP regulatory TMC formation with active FI at 25°C, 118nM of FH1-4 and 118nM WT (active) FI were injected simultaneously.

To test AP regulatory TMC formation with I340T or H380R FI, 125nM of FH1-4 and 125nM I340T/H380R FI were injected separately and simultaneously.

All TMC building tests were repeated at least twice, with variants compared a minimum of once on thioester-coupled (shown in main text Figures) and once on amine-coupled (shown in **Supplemental Data**) CM5 chip surfaces.

#### Dominant Negative SPR experiment

Inactive WT (S525A) FI (125nM) was injected with WT FH1-4 (125nM) to form a WT TMC as standard as described earlier. To determine the dominant negative effect, I340T FI was injected at 250nM with inactive WT FI and WT FH1-4 (both at 125nM).

### Haemolytic sheep red blood cells (SRBC) cofactor assay

Haemolysis assays were performed as per those described in Tortajada et al. (29) with some modification. FI variants were titrated 1:2 8 times in a 96-well plate in complement fixation diluent (CFD) [supplemented with 2µg/mL FH (CompTech)] before incubation with C3b-coated SRBC for 10 mins at 37°C. Washing and incubation with FB (20µg/mL) and FD (2µg/mL) followed by further washing and incubation with 2% Guinea pig serum in 10mM EDTA resulted in lysis of the cells dependent on FH1-4 concentration. A 0% lysis control with no FD/FB incubation was used to normalise and give % protection plotted. Data is representative of 3 experimental repeats performed in duplicate.

### Statistical analysis

All statistical analysis presented herein was performed using GraphPad Prism V8 (GraphPad Software, La Jolla, CA, USA). For fluid phase CA assays, data were expressed as means +/- SD and standard t-tests were used to identify any differences in activity between variants and the WT protein. For haemolytic SRBC assays mean +/- SD for individual points is plotted by bars and IC50s with 95% confidence intervals were calculated using the ‘normalised response (4 parameter fit)’ non-linear model.

## Results

### Generation of fully processed factor I

**Figure 2:**
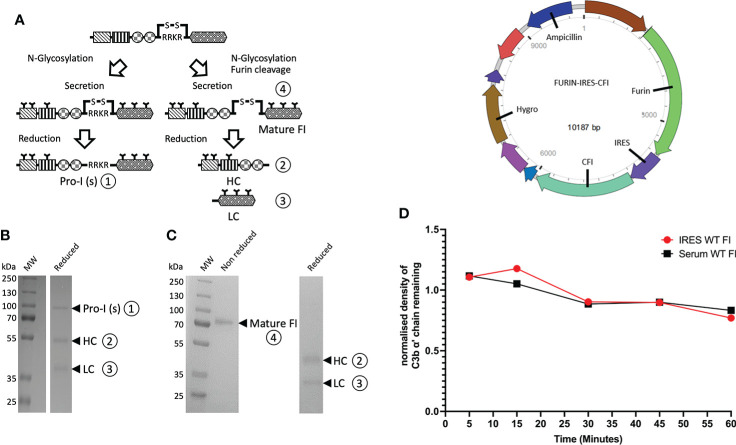
Utilisation of an IRES vector to produce fully processed FI in mammalian cells. **(A)** FI is synthesized as a single polypeptide chain precursor [pro-I]-81 kDa. Upon translocation to the endoplasmic reticulum, and prior to secretion, pro-I undergoes post-translational processing involving cleavage at its linking peptide (RRKR) and N-glycosylation of both chains. In mammalian cells transfected with a vector containing only the *CFI* gene, not all of the pro-I undergoes cleavage of the RRKR linker peptide. This results in secretion of an N-glycosylated pro-I [pro-I(s)] ① of 88 kDa and a mature disulphide-linked FI ④ consisting of a 50 kDa heavy chain (HC) ② and a 38 kDa light chain (LC) ③. The incomplete cleavage of the pro-I form is thought to be due to the high expression of the protein that saturates the cleaving ability of the cells. **(B)** Reduced SDS-PAGE of historic generation of recombinant FI demonstrating a mixture of uncleaved inactive Pro-I (s) ① and the HC② and chain ③ of fully processed mature FI. **(C)** Reduced and non-reduced SDS-PAGE gel of recombinant FI using our optimised *Furin-IRES-CFI* vector demonstrating only mature FI ④ with no Pro-I(s) on the reduced gel. **(D)** A map of the *Furin-IRES-CFI* vector by VectorBuilder: https://en.vectorbuilder.com/vector/VB171219-1127wqz.html. **(E)** C3b α’ chain degradation analysis of the fluid-phase activity of IRES WT FI compared to serum purified FI, over time. Plotted is the density of C3b α’ chain remaining (y-axis) after incubation with recombinant or serum purified FI with C3b and FH across a range of timepoints (5 – 60 minutes) at 37°C during a fluid-phase cofactor assay. The density of the α’ chain band was normalised to the density of the β chain band, before the resultant Figure was normalised to a negative control containing no FI, giving a proportion of α’ chain remaining compared to the no FI control.

**Figure 3:**
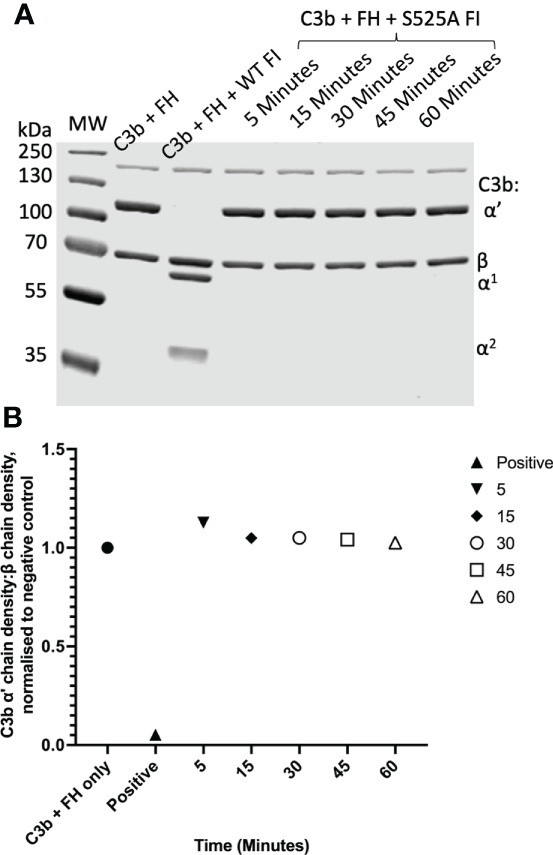
Fluid phase analysis of inactive (S525A) FI. **(A)** SDS-PAGE demonstrates a lack of proteolytic activity of IRES S525A FI across a range of timepoints (5 – 60 minutes). Activity was assessed by the ability of factor I in combination with its cofactor, FH, to cleave C3b to its inactive form, iC3b. C3b cleavage was indicated by the generation of the α1 (68kDa) and the two α2 (46kDa, 43kDa) bands as demonstrated by the WT FI (positive) control. **(B)** Lack of activity of S525A FI – densitometry analysis. Licor ImageStudio Lite with median background subtraction was utilised to perform densitometry analysis on the SDS-PAGE gels revealing no activity of the S525A FI. The density of the α’-chain was given as a ratio of the β-chain density before being normalised to a negative control (with no FI) to give a relative density of the α’-chain remaining for each reaction.

Fully processed FI with no evidence of unprocessed pro-I on SDS-PAGE (**Figures 2A–C**) was generated using an IRES vector containing *Furin* and *CFI* genes (**Figure 2D**). The recombinant FI generated using the *Furin-IRES-CFI* vector demonstrated equivalent activity to FI purified from human serum (**Figure 2E** and **Supplemental Figure 1**). Using this template an inactive FI variant was generated for use in real-time AP TMC regulatory complex analysis. Mutation of the proteolytic serine residue S525 to an alanine (S525A FI) prevented proteolysis of C3b (25) (**Figures 3A, B**; **Supplemental Figure 2**).

### Variant selection and protein preparation

Rare variants in *CFI* and *CFH* associated with AMD (**Supplemental Tables 3, 4**) [FI: I340T (13, 20, 30–32), R406H (13, 16, 30, 31), K441R (13, 16, 30, 31), P553S (13, 16, 30, 31) and FH CCP 1-4: P26S (33), T91S (33), R166W (33), R232Q (33)] were modelled within the context of the AP regulatory TMC (25) (**Figure 1**). FH variants were generated in the framework of the N-terminal domains of FH, CCPs 1–4, as previously described (34) (**Supplemental Figure 3A**). FI variants were produced using the *Furin-IRES-CFI* vector (**Supplemental Figures 3B, C**). Additionally, we generated R83S *CFH*, which is a highly deleterious C3 glomerulopathy (C3G)-associated non-functional variant that was used a positive control for dysfunction (27) and H380R *CFI*, which was identified in a patient with clinical *CFI* deficiency (35) and was predicted to be inactive due to mutation of the FI active site (36).

### Rare AMD -linked *CFH* Variant characterisation

**Figure 4:**
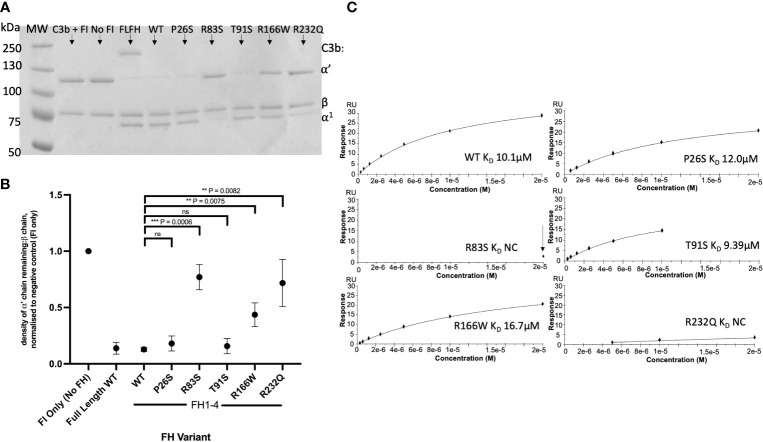
Characterisation of AMD –linked FH CCP 1-4 Variants. **(A)** Fluid phase cofactor assays for FH1-4 variants. Separation of FI -cleaved C3b products by SDS-PAGE followed by Coomassie staining was used to reveal the α’, β and α1 chains of C3b. **(B)** Densitometry analysis of C3b breakdown by each FH1-4 variant. The mean (+/- SD) density of the normalised α’-chain present in the products of 3 fluid phase reactions is plotted for each FH 1-4 variant. The density of the α’-chain was given as a ratio of the β-chain density before being normalised to a negative control (with no FH CCP 1-4) to give a relative density of the α’-chain remaining for each reaction. Higher (closer to 1) α’-chain remaining suggests reduced cofactor activity of the FH CCP 1-4 variants. A standard t test was used to compare mean normalised α’-chain remaining values for each variant vs the WT. **P < 0.01; ***P < 0.001. ns = non-significant. **(C)** 20µM (and 6 subsequent 1:2 serially diluted concentrations) of each FH CCP 1-4 variant and WT was injected onto a C3b –coupled CM5 chip (800-1000RU) to give an estimated K_D_ binding affinity of each variant to C3b after analysis by SPR using a BIAcore S200. SPR Figures are representative of at least 2 repeats.

CCP1-4 FH variants were characterised by standard assays prior to analysis of TMC formation. Fluid phase cofactor activity (CA) assays with FI and C3b revealed observable (**Figure 4A**) and statistically significant (**Figure 4B**) reductions in C3b α’ chain degradation by R83S (P = 0.0006), R166W (P = 0.0075) and R232Q (P = 0.0082) (64%, 58% and 41% reduced compared to WT, respectively). Meanwhile, P26S and T91S had similar fluid phase CA to the WT protein (4% and 3% reduced compared to WT, respectively).

Variants were further analysed by measurement of affinity (*K*_D_) to C3b using surface plasmon resonance (SPR). Assays were performed twice, once in HBST and once in PBST (PBST shown). The WT showed similar C3b binding affinity to FH CCP1-4 to previous publications, with an approximate *K*_D_ of 10µM (26, 27, 37). P26S and T91S bound C3b with similar affinity to WT (*K*_D ~_11.5 and _~_9.4 µM, respectively) whilst R166W showed reduced affinity (_~_16.7µM). R83S and R232Q were broadly abrogated in C3b binding even at high (20µM) concentrations and as such their C3b affinities were designated as not calculable (NC) (**Figure 4C**).

**Figure 5:**
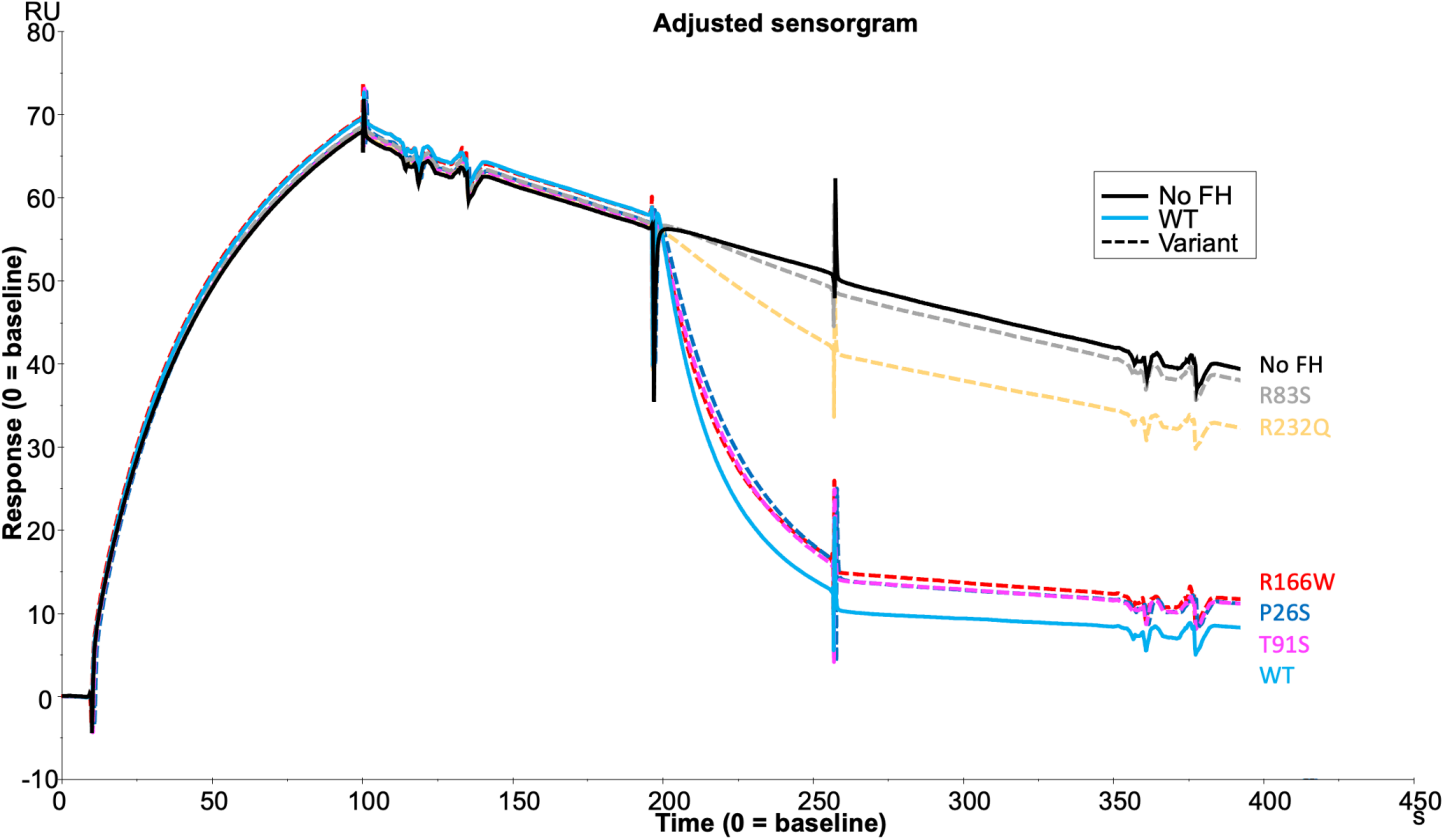
DAA Assay for AMD -linked FH CCP1-4 Variants. C) Each FH CCP 1-4 variant and WT was injected at 250nM onto a C3b -coupled CM5 chip (800RU) after building of the AP C3 convertase to reveal the DAA of each variant compared to WT and a no FH1-4 injection as measured by SPR using a BIAcore S200. SPR Figures are representative of at least 2 repeats.

Furthermore, measurement of decay accelerating activity (DAA) by SPR consistently revealed that R83S and R232Q had a near total loss of DAA of the AP C3 convertase consistent with their lack of binding to C3b. Meanwhile, R166W, P26S and T91S had only minor reductions in function in the DAA assay (**Figure 5**). Strikingly, R166W showed disproportionately reduced CA compared to DAA.

#### Real-time analysis of FH variants in formation of the AP regulatory tri-molecular complex

**Figure 6:**
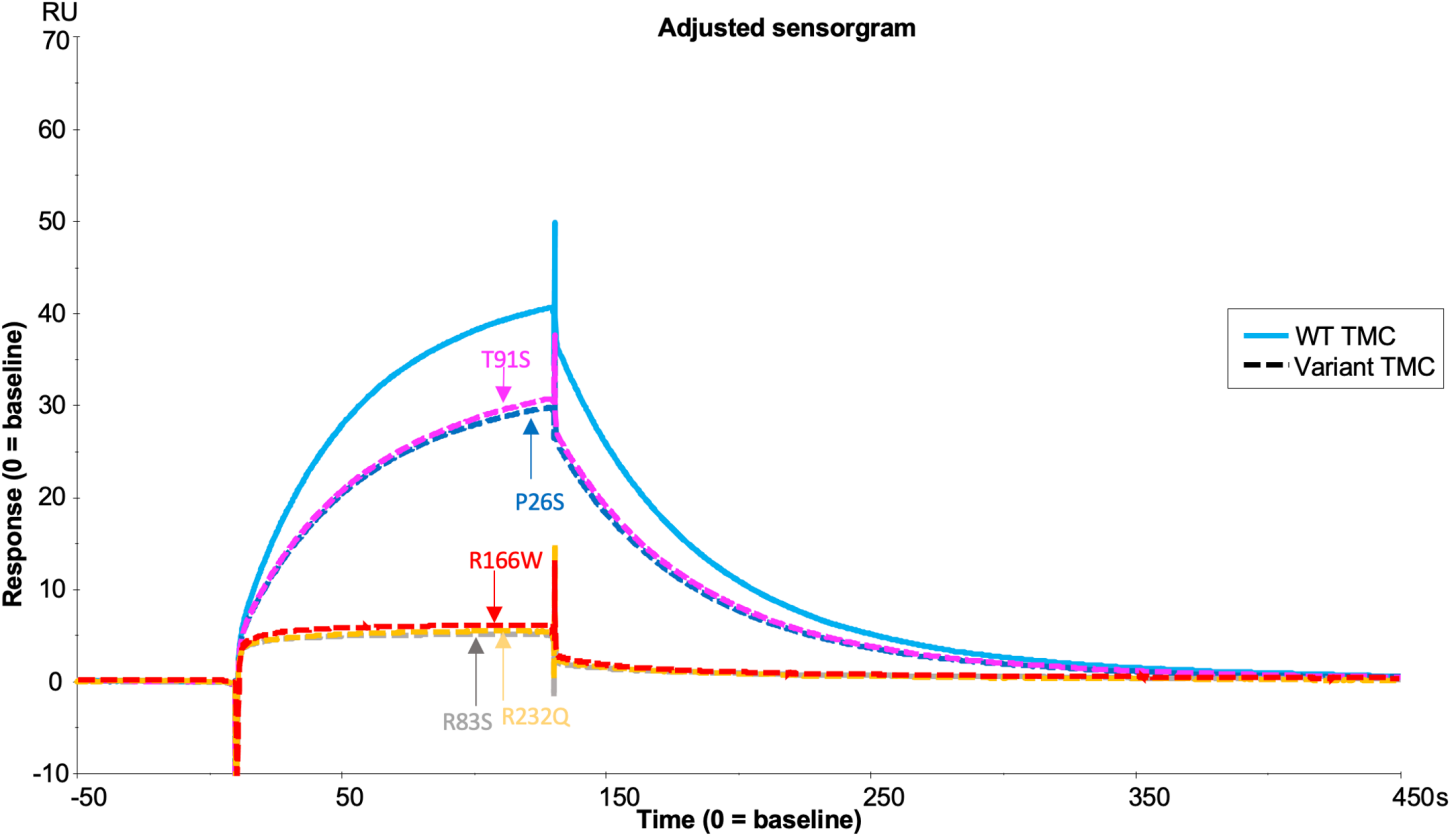
TMC building SPR assay for FH1-4 variant analysis. Each FH CCP 1-4 variant was injected at 59nM with 118nM of S525A FI onto a C3b (1000RU) thiol –coupled CM5 chip surface and analysis by SPR revealed real-time binding of the AP regulatory TMC for each variant and the WT. SPR Figures are representative of at least 2 repeats.

Assessment of the formation of the AP regulatory tri-molecular complex using SPR is complicated by cleavage of the substrate C3b by active FI protein (**Supplemental Figure 4**). To overcome the issue of substrate cleavage, the inactive (S525A) FI variant was used in the formation of the AP regulatory TMC to facilitate real-time analysis of the effect of FH variants on TMC formation as measured by SPR (**Figure 6**). Injections of WT FH CCP1-4 with inactive FI onto a C3b thiol coupled surface revealed the formation of synergistic complexes; these were larger and long lasting compared to the bi-molecular complexes formed by C3b and FH1-4 (**Supplemental Figure 5**). The results were consistent with the earlier standard CA assays with 59nM injections of R166W (7RU), R232Q (6RU) and R83S (5RU) displaying minimal TMC formation compared to WT, whilst P26S (29RU) and T91S (30RU) generated peak RUs of approximately 75% that of WT (40RU) TMC.

Comparison of FH variant TMC formation efficacy was equivalent when C3b was tethered by amine coupling (**Supplemental Figure 6**) or covalently bound through nucleophilic attack on the internal thioester of nascent C3b. Although responses were higher and the orientation physiological in the thiol-coupled method, C3b bound to the chip *via* targeting of its thioester bond is hydrolysed in a time-dependent manner. Therefore, the AP convertase, C3bBb, was generated at the start and end of each experiment as a measurement of surface stability showing ~12% loss of surface activity (**Supplemental Figure 7A**). The amine coupled surface, however, exhibited no loss of C3b between the first convertase injection and the last during an otherwise identical experiment (**Supplemental Figure 7B**).

### Rare AMD-linked *CFI* Variant Characterisation

**Figure 7:**
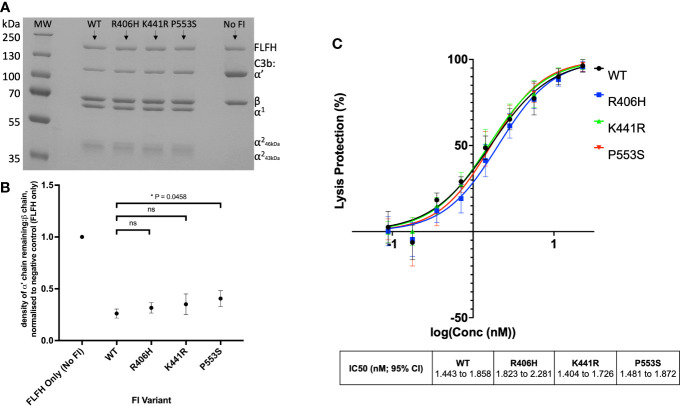
Characterisation of AMD –linked CFI Variants. **(A)** Fluid phase cofactor assays for FI variants. Separation of C3b products by SDS-PAGE followed by Coomassie staining was used to reveal the α’, β and α1 chains of C3b. **(B)** Densitometry analysis of C3b breakdown by each FI variant. The mean (+/- SD) density of the normalised α’-chain present in the products of 3 fluid phase reactions is plotted for each FI variant. The density of the α’-chain was given as a ratio of the β-chain density before being normalised to a negative control (with no FI) to give a relative density of the α’-chain remaining for each reaction. Higher (closer to 1) α’–chain remaining suggests reduced cofactor activity of the FI variants. Fluid phase assays were repeated 3 times and a standard t test was used to compare mean normalised α’-chain remaining values for each variant vs the WT FLFH = full-lenght factor H. *P < 0.05. ns = non-significant. **(C)** Haemoltyic assays of CA for FI variants. Fi variants were titrated through C3b-coated SRBCs before the AP C3 convertase was built on any C3b remaining on the cell surfaces. Cells were lysed with Guinea pig serum in an FI concentration dependent manner with a readout of OD at 412nm. The efficacy of each FI variant was calculated using a non-linear 4-parameter fit curve after normalization to no FI (100%) and 0% (buffer only) lysis controls and IC50s are given with 95% CIs. Each plotted point is % protection from lysis compared to the 0% lysis control and bars represent SD for each point calculated from 3 experimental repeats run in duplicate.

Fully processed FI variants (with no pro-I) were first characterised using fluid phase CA assays measuring proteolytic cleavage of the C3b α’ chain. These fluid phase assays revealed similar levels of activity for R406H (5.5% reduced) and K441R (9% reduced) compared to WT, whilst P553S (14.4% reduced vs WT, P = 0.046) had significantly reduced C3b cleavage with full length FH as the cofactor (**Figures 7A, B**). The fluid phase CA assay data were corroborated by a haemolytic C3b cofactor assay on sheep erythrocytes, demonstrating that WT, K441R, R406H and P553S had similar ability to protect C3b-coated sheep RBC from complement AP mediated lysis, albeit that R406H and P553S exhibited minor non-significant reductions in efficacy (**Figure 7C**).

#### Real-time analysis of FI variants in formation of the AP regulatory trimolecular complex

**Figure 8:**
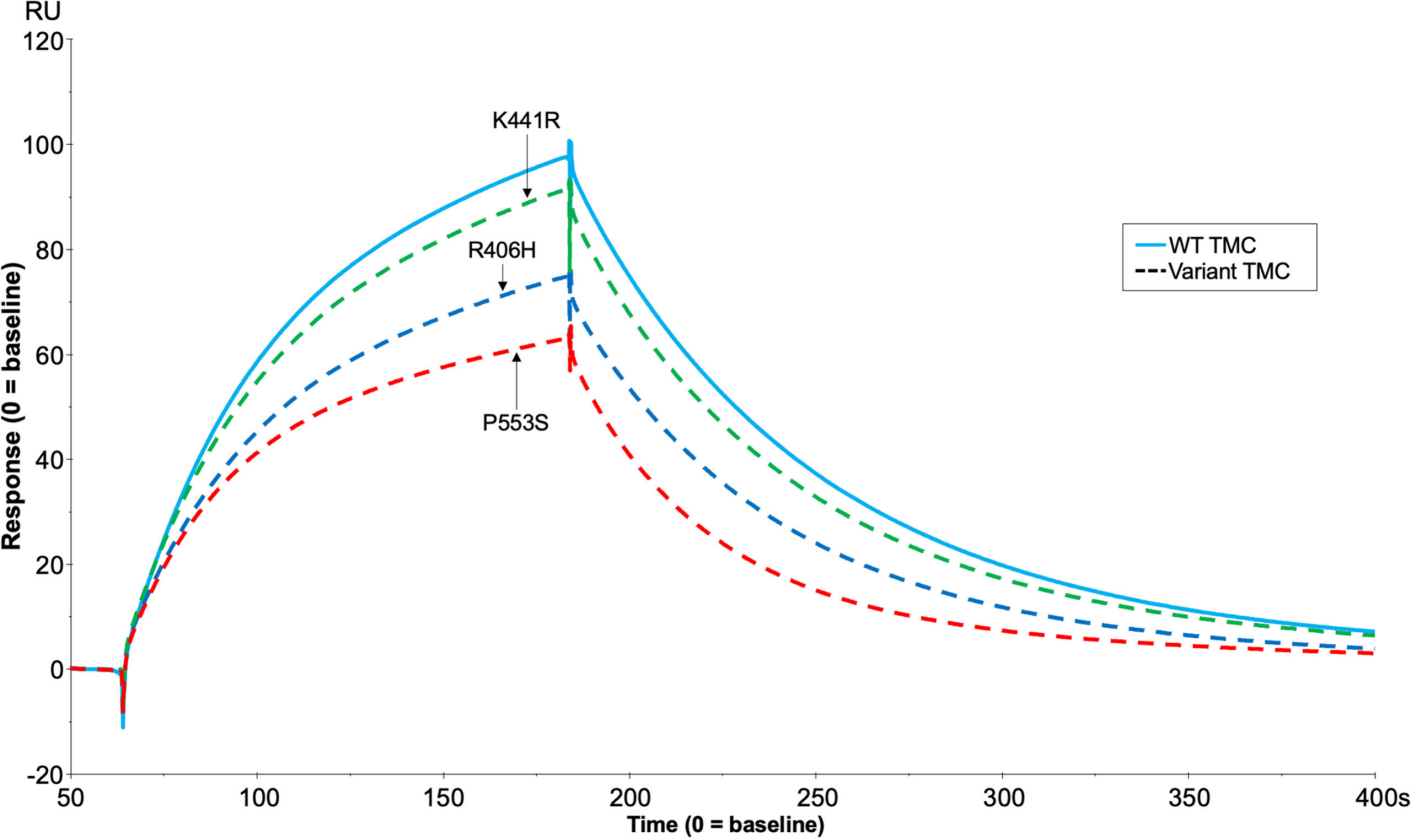
TMC building SPR assay for FH1-4 variant analysis. Each inactive FI variant (S525A+R406H/K441R/P553S/WT) was injected at 62.5nM with 118nM of FH CCP1-4 onto a C3b (1000RU) thiol -coupled CM5 chip surface and analysis by SPR revealed real-time binding of the AP regulatory TMC for each variant and the WT. SPR Figures are representative of at least 2 repeats.

Analysis of the TMC is hindered by the FI variants possessing C3b-cleaving activity (e.g. R406H, K441R, P553S) as the complex rapidly dissociates following cleavage. To circumvent this, we generated the FI variants on an inactive (S525A) FI variant backbone (e.g. double mutant proteins: R406H/S525A; K441R/S525A; P553S/S525A). This facilitated real-time visualisation of TMC formation for each FI variant versus the inactive WT (e.g. WT/S525A) (**Figure 8**). When the TMC was assembled on a C3b thioester-coupled surface, inactive P553S formed substantially less TMC compared to the inactive WT FI protein as measured by SPR after injection at 62.5nM (62.9 RU, 65% of WT). Inactive R406H showed a minor reduction in efficacy in this assay (74.6 RU, 77% of WT), akin to the effects of P26S and T91S in the FH1-4 variant analysis. Meanwhile, K441R TMC building activity was almost identical to the WT inactive protein (91.2 RU, 94% of WT). These findings agreed with fluid phase and haemolytic assay data and the trend was also replicated when building TMCs on an amine C3b -coupled surface (**Supplemental Figure 8**).

### Dominant Negative effect of *CFI* Variants in fluid phase cofactor assays

**Figure 9:**
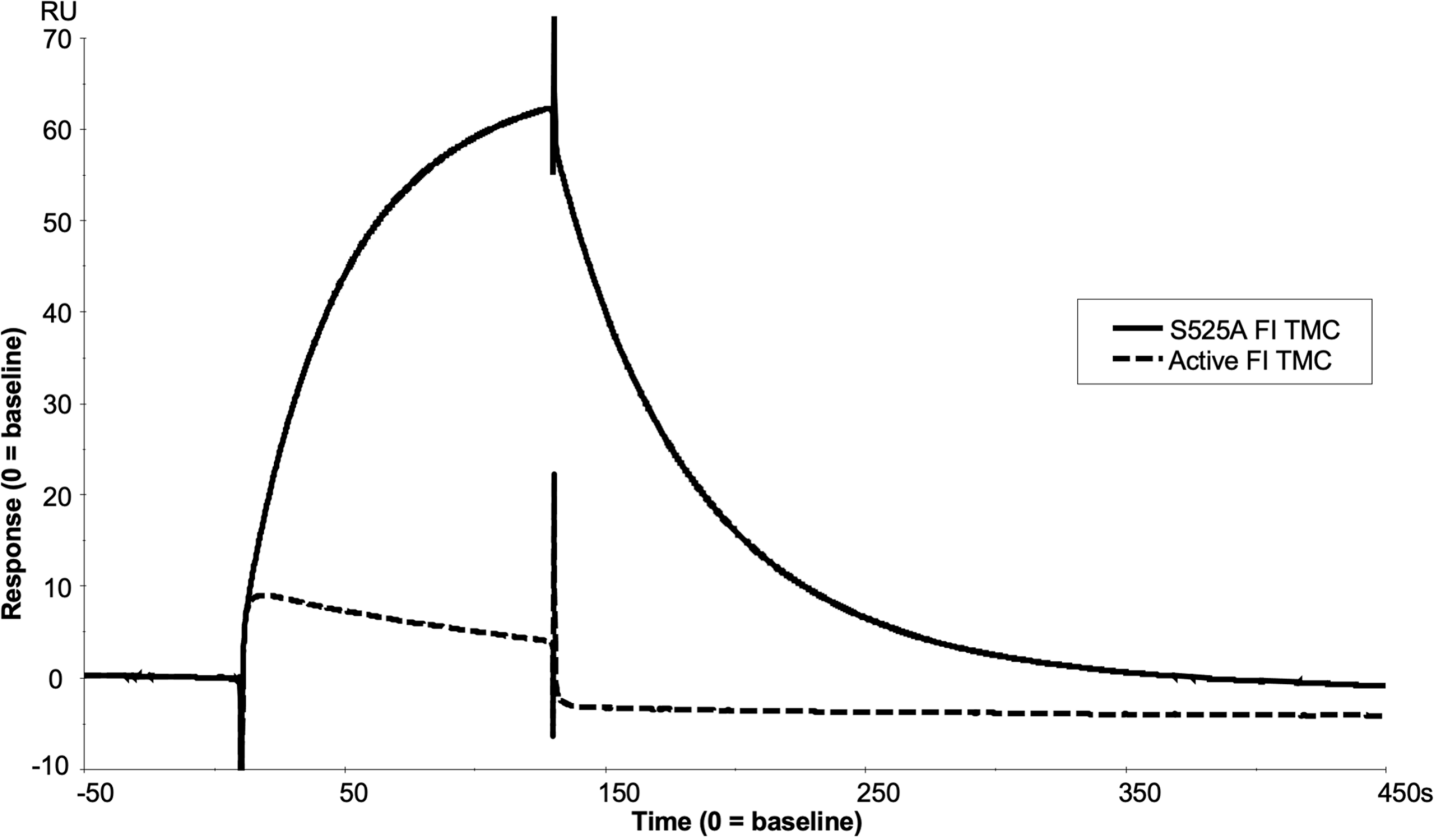
SPR analysis of the inactive FI variant vs active FI. Injection of 118nM of active FI with 118nM of FH1-4 onto a C3b thiol coupled (1000RU) CM5 chip resulted in near immediate loss of C3b on the chip surface as shown by a downward trend in RU vs time (dashed line) upon injection, exhibiting loss of FH1-4 binding affinity to iC3b vs C3b. Meanwhile, injection of 118nM of inactive FI and 118nM of FH1-4 onto the same surface resulted in the building of a comparably large AP regulatory TMC (of ~60RU) (solid line) which did not immediately dissociate when the injection finished at 120 seconds.

**Figure 10:**
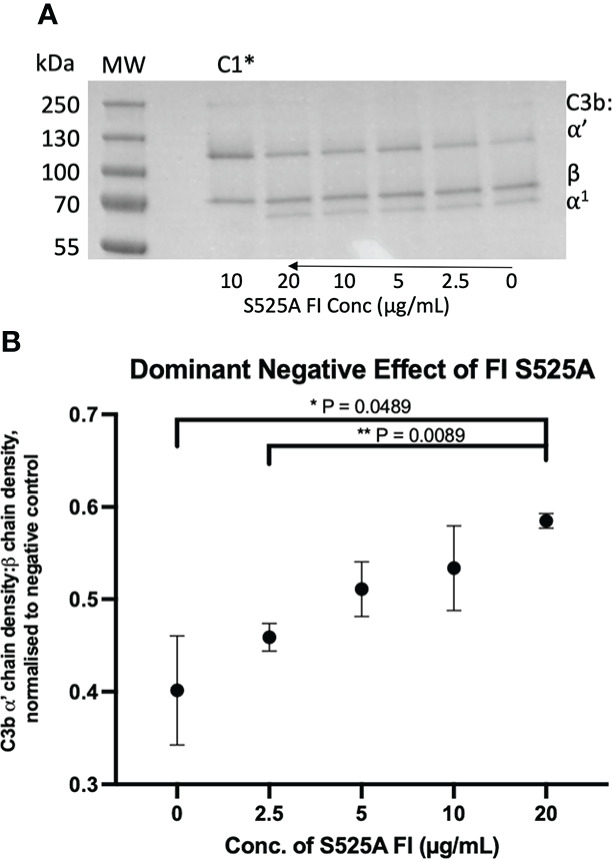
Identification and characterisation of a dominant negative effect in a model inactive FI variant. **(A)** Fluid phase assay revealed a dominant negative effect of inactive (S525A) FI on active FI. Addition of 5µL of 0, 2.5, 5, 10 and 20 µg/mL inactive FI to a standard FI cofactor assay before incubation resulted in an inhibitory effect as shown by lower levels of breakdown of the C3b α’-chain when 20µg/mL inactive FI was spiked compared to a 0µg/mL control lane. *C1 = Control 1 (negative control including C3b, FH1-4, S525A and no WT FI. **(B)** S525A dominant negative effect – densitometry analysis. Licor ImageStudio Lite with median background subtraction was utilised to perform densitometry analysis on the SDS-PAGE gels resulting from 2 individual repeats of the S525A dominant negative fluid phase assay described. Graphpad prism V8 was utilised to test for differences between tests and mean +/- SD is plotted. *P < 0.05; **P < 0.01. C1 = control 1 [C3b, FH1-4 and S525A FI only (no WT FI)].

Real-time SPR analysis demonstrated that inactive FI (S525A) formed stable, long lasting C3b:FH:FI complexes compared to active FI (**Figure 9** and **Supplemental Figure 9**). This implied that secreted inactive variants in *CFI* may cause a competitive inhibitory effect on the WT FI protein. To test this hypothesis, increasing concentrations of S525A were added to WT FI in a fluid phase CA assay demonstrating a concentration-dependent inhibition of WT activity. Cleavage was reduced by 15% at equimolar concentrations, and by 20% at 2-fold molar concentrations. (**Figures 10A, B**).

Although S525A is not a reported mutation in patients, a review of patient associated mutations revealed several variants that would be predicted to have a similar effect: I340T [AMD (31), atypical haemolytic uraemic syndrome (aHUS) (20)] and H380R [functional FI deficiency (35)], D519N and D524 [aHUS (20)]. Two variants, I340T and H380R, were taken forward for further analysis due to their existence in AMD and topology within the FI active site, respectively.

**Figure 11:**
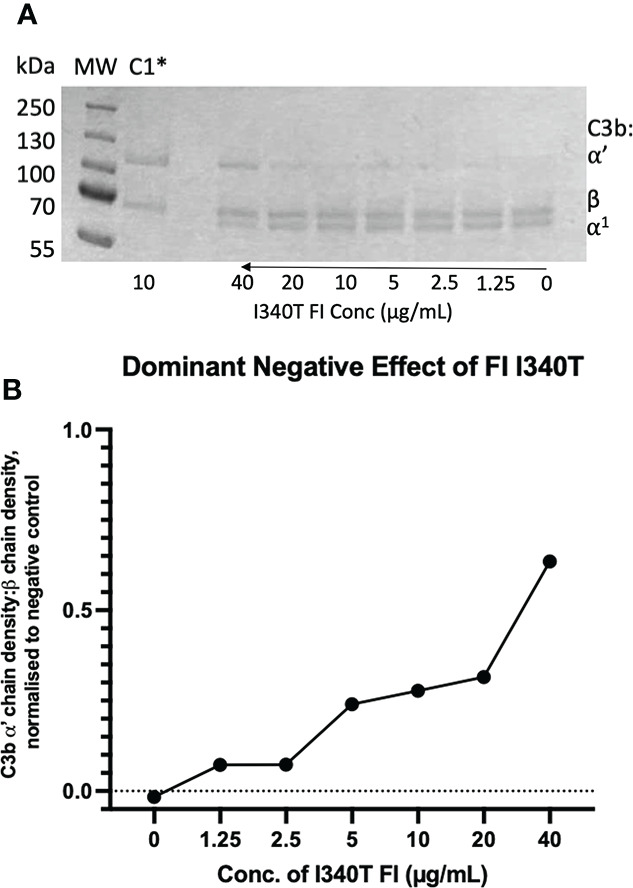
Identification and characterisation of a dominant negative effect in an AMD -linked FI variant. **(A)** Fluid phase assay revealed a dominant negative effect of I340T FI upon active WT FI. Addition of 5µL of 0, 1.25, 2.5, 5, 10, 20 and 40 µg/mL I340T FI to a standard FI cofactor assay before incubation resulted in an inhibitory effect as shown by lower levels of breakdown of the C3b α’-chain when 40 µg/mL inactive FI was spiked compared to a 0 µg/mL control lane. *C1 = Control 1 (negative control including C3b, FH1-4, I340T and no WT FI. **(B)** I340T dominant negative effect – densitometry analysis. Licor ImageStudio™ Lite with median background subtraction was utilised to perform densitometry analysis on the SDS-PAGE gel resulting from the I340T dominant negative fluid phase assay described. Plotted are densities of C3b ⍺’ -chain remaining normalised to the beta chain for each test and normalised to the negative control (C1) value. Graph produced using GraphPad Prism V8. C1 = control 1 [C3b, FH1-4 and I340T FI only (no WT FI)].

Neither I340T nor H380R demonstrated any enzymatic activity in fluid phase CA assays, as shown in “C1” lanes in **Figure 11A** and **Supplemental Figure 10A**, respectively. When increasing concentrations of I340T or H380R were added to WT FI in a fluid phase CA assay, competitive inhibition was demonstrated. I340T was shown to inhibit the activity of the WT protein by ~25% at equimolar concentrations, and by 30% at 2-fold molar concentrations (**Figure 11B**). H380R was shown to inhibit the activity of the WT protein by ~20% at equimolar and 35% at 2-fold molar concentrations (**Supplemental Figure 10B**). An additional test for non-specific protein interference in fluid phase kinetics was performed using human serum albumin (HSA); and no concentration-dependent blocking effect was demonstrated with this serum-abundant protein (**Supplemental Figure 11**). These data provide evidence that the inactive FI dominant negative effect is a specific effect of FI interaction with C3b and/or FH that does not result in cleavage of C3b but does competitively inhibit the activity of WT FI.

### Real-time analysis of TMC formation in the presence of disease-linked inactive FI variants

**Figure 12:**
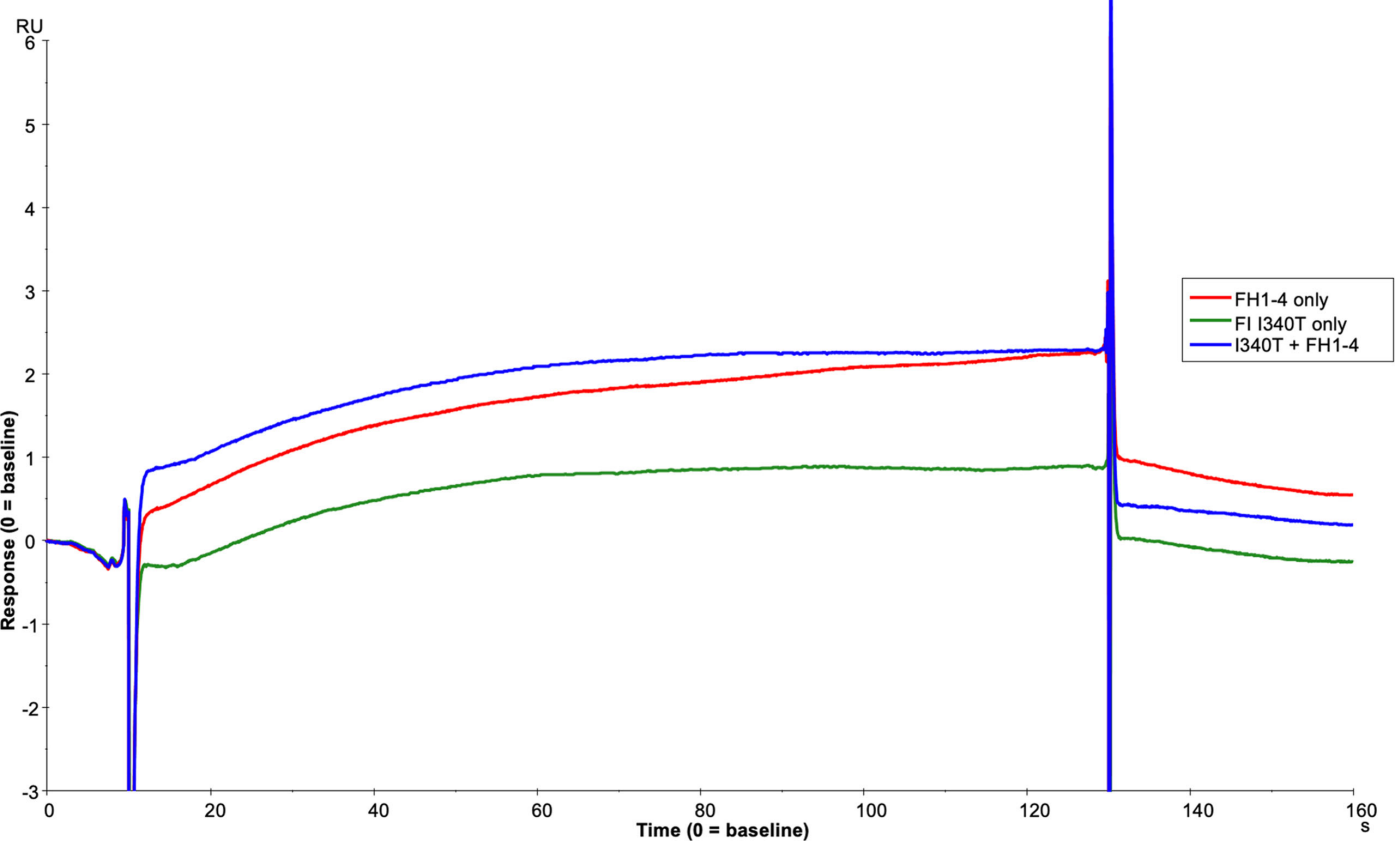
I340T does not form a TMC with C3b and FH CCP1-4. Using the BIAcore S200, FH1-4 (red line) and I340T FI (green line) were injected at 125nM individually and in combination (blue line) onto a 1000RU C3b coupled CM5 chip surface. I340T FI did not display synergy with FH1-4 indicating no or minimal formation of the AP regulatory TMC.

**Figure 13:**
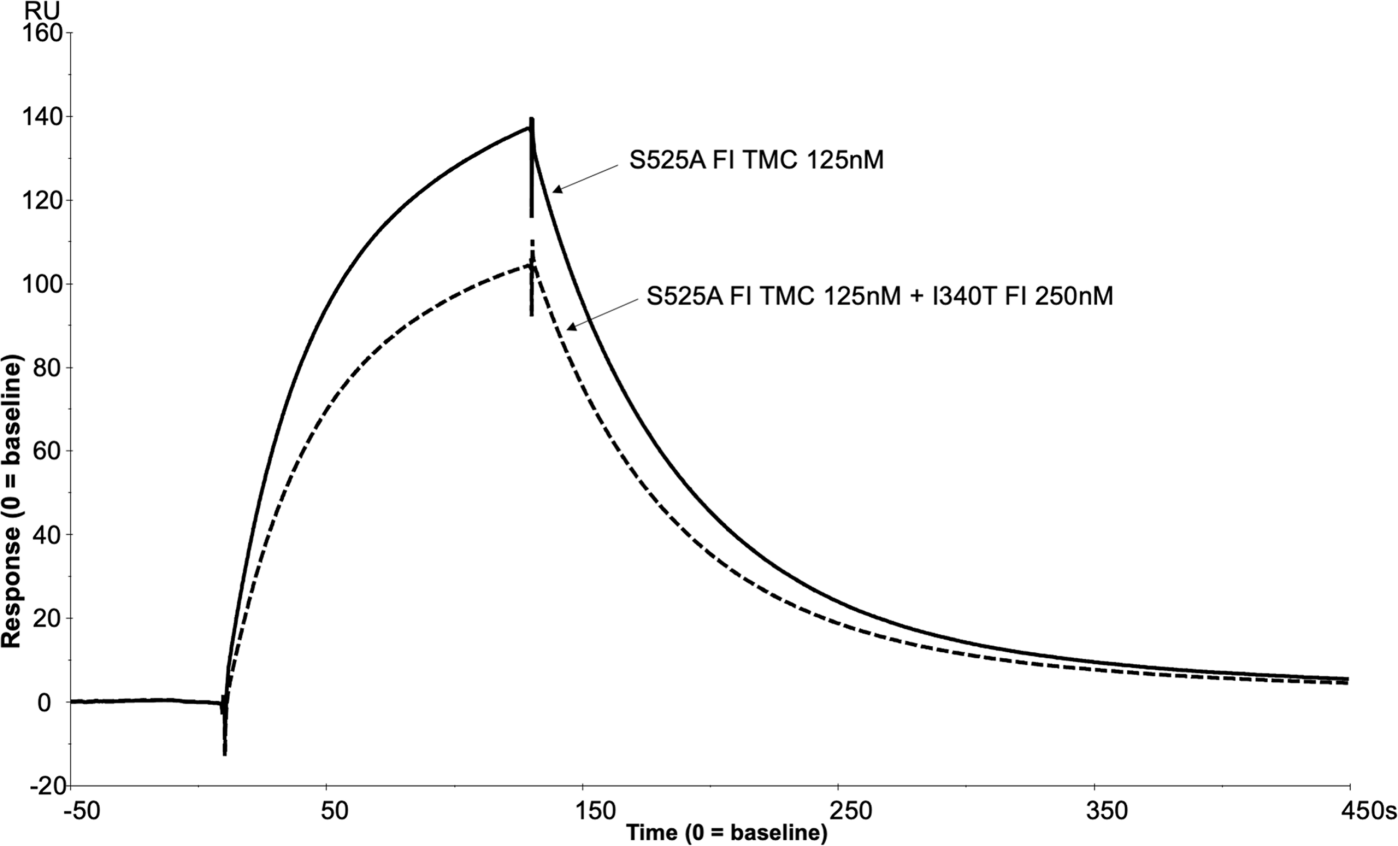
SPR reveals I340T FI inhibits formation of the AP regulatory TMC. To build an AP regulatory TMC 125nM of inactive WT FI and 125nM of FH CCP 1-4 was injected onto a C3b thiol coupled (1000RU) CM5 chip (solid line). A further TMC was built by injection of 125nM of Inactive WT FI, 250nM of I340T FI and 125nM of FH CCP 1-4 onto the same thiol coupled chip (dotted line). Addition of the I340T FI protein resulted in an ~25% lower maximum RU compared to inactive WT FI only as measured by SPR on the Biacore S200. SPR assays are representative of at least 2 independent experiments.

To further assess the biological impact of the inactive FI variants, SPR was undertaken with single mutants. As with the mutant S525A, the patient associated mutation, H380R, could bind to C3b:FH complexes without cleavage of the surface bound substrate, explaining its competitive inhibition of WT active FI (**Supplemental Figures12**). However, unlike with H380R and S525A FI, when I340T was injected with FH1-4 there was no evidence of a TMC being formed (**Figure 12**), yet when I340T FI alone was injected at higher concentrations (350nM) onto a C3b coupled surface, some direct interaction with C3b was observed (**Supplemental Figure 13**). Further, addition of I340T at 2-fold molar concentration [vs S525A FI (125nM)] during formation of WT inactive (S525A) TMC resulted in ~25% lower maximum RU in keeping with the CA assays and confirming the competitive effect (**Figure 13**).

### Wider applicability of the TMC building assay

AP regulatory TMCs were also assembled on the SPR chip with FH-like protein 1 (FHL-1), soluble complement receptor 1 (sCR1, CD35) (TP10) and membrane cofactor protein (MCP, CD46) (**Supplemental Figures 14, 15**). This opens the possibility to interrogate the functional impact of variants in these other cofactors which will be important in other complement mediated diseases such as paroxysmal nocturnal haemoglobinuria (38), aHUS (39) and C3G (40).

## Discussion

The ability to produce recombinant fully processed FI is a significant methodological advance which underpins this study. Recombinant FI production has historically yielded a mixture of active FI and inactive pro-I (20, 41) with co-transfection of cells with furin and FI vectors ineffective (41) and ex-vitro cleavage of pro-I inefficient (42).

In AMD, where the manifestation of disease may take decades, the effects of rare genetic variants may be small. Variations in the amount of inactive pro-I contaminating protein preparations may therefore mask small defects in activity. The *Furin-IRES-CFI* vector circumvented these problems, yielding completely processed FI with equivalent activity to serum-purified protein. Additionally, the ability to make fully processed FI allowed us to produce an inactive, fully processed variant to generate a real-time analysis of the AP regulatory TMC to view the activity of variants in FH and FI. The use of SPR to visualise the AP regulatory TMC represents huge potential for analysis of functional effects of AP protein variants.

In analysis of AMD-linked *CFH* variants (summarised in **Supplemental Table 5**), marked dysfunction in AP regulation was observed in 2 variants: one in CCP 3 (R166W) and another in CCP 4 (R232Q) (25, 43, 44). R232Q resides in CCP 4 and forms part of a contact region with macroglobulin (MG) 1 and thioester (TED) domains of C3b. It is highly deleterious, abrogating both CA and DAA due to impaired C3b binding and consequently a failure to form the regulatory TMC. R232Q replicates the effects seen in the C3G-associated R83S variant which likewise perturbs a contact patch between C3b and the terminal region of FH CCP 1 and the CCP 1-2 linker to the α′ N-terminal region and MG7 domain in C3b.

In contrast to R232Q, R166W sits in the third contact site with the CUB domain of C3b but also at the interface with the SP domain of FI (25, 43, 44). Its affinity for C3b was affected mildly (16-20µM vs 10µM in the WT protein) with a minor decrease in DAA. In comparison, CA activity was markedly reduced and TMC formation was profoundly affected with the slightly higher RU compared to R83S and R232Q reflecting the maintained ability of R166W to bind C3b.

The remaining 2 variants, P26S and T91S, displayed only minor differences in CA, DAA and TMC building, exhibiting levels of defect similar to the common I62V risk SNP for AMD (26, 29). As both P26S and T91S are solvent exposed and not predicted to interact directly with either FH or FI, any effect is likely due to minor steric alteration introduced by the variants (25, 43, 44).

Three analysed AMD associated FI variants (R406H, K441R and P553S) demonstrated the ability to cleave C3b (as summarised in **Supplemental Table 5**). Fluid phase CA assays demonstrated non-significant reductions in C3b cleavage efficacy by R406H and K441R, while P553S demonstrated a significant, albeit small, reduction compared to WT FI. Our real-time assessment of these variants on an inactive FI backbone correlated closely with the CA assay results. P553S demonstrated the largest reduction in TMC formation and R406H and K441R demonstrated less marked defects. Despite the impaired binding and activity, P553S does not interface with either C3b or FH and is distinct from the catalytic triad (25, 45). P553S resides in a disordered loop in free FI which is stabilized upon formation of the TMC, a process that may be perturbed by the variant impacting substrate binding. The R406H variant is also in an unstructured loop in free FI and when complexed, is at an interface between FH and FI interacting with E123 in CCP 2 of FH suggesting a stabilizing role of this interaction (25, 45). The K441R variant has the least impact of any of the variants analysed, despite a predicted interaction with N136 in CCP 2 of FH (25, 45). The K441R variant does not result in a substantial alteration in charge or size of the side chain at position 441, which may explain this lack of impact.

Previous functional analysis of P553S, R406H and K441R using serum assays and recombinant protein has been contradictory (20–22, 46) likely due to an inability to adequately quantitate mutant vs wild type proteins and/or the level of contamination with Pro-I (20, 21). The real-time TMC binding assay described herein demonstrates that minor alterations in CA at the surface or in the fluid phase can be deciphered by SPR. These data suggest this new method is highly sensitive to changes in the binding efficacies of FI variants to C3b:FH complexes. That predisposing polymorphism in C3, FB and FH have small additive effects (47) reinforces the requirement for accurate analysis of variants.

Additionally, this TMC formation assay has allowed us for the first time to demonstrate dominant negative effects (48) of FI mutants described in AMD, aHUS and functional FI immunodeficiency.

The H380R FI variant was detected in a patient with a clinical complement deficiency (35). H380 along with D429 and S525 form the catalytic triad of FI. With the catalytic site mutated, H380R FI could not cleave C3b and was completely inactive. This allowed the variant to be used in TMC building experiments (without mutating onto an S525A backbone) demonstrating formation of the regulatory complex. Because the SPR analysis shows a slow on, slow off binding pattern, the inactive complexes may exist for considerably longer than active complexes, which appear to cleave C3b instantaneously and destabilise the complex (**Figure 9** and **Supplemental Figure 9**). The fact that H380R FI could readily bind C3b:FH complexes without cleaving C3b, suggested competitive inhibition of WT FI was possible, and this was demonstrated using a CA assay (**Supplemental Figure 10**). This dominant negative effect would have no consequence in the homozygous proband presenting with immunodeficiency but would be predicted to impair WT function in heterozygous relatives.

The I340T FI variant, which has been identified in aHUS (20) and AMD cohorts (16) also demonstrated a dominant negative effect in fluid phase CA assays. Unlike H380R however, I340T showed little evidence of TMC formation but it did bind C3b and displayed the ability to inhibit WT FI in TMC formation. This is due to loss of the isoleucine that stabilises an oxyanion hole at a catalytic triad interaction site on C3b. Thus, we demonstrated that secreted variants distinct from the catalytic triad which have impaired CA activity but retain the ability to interact with C3b and/or FH, have the capability to competitively inhibit WT FI.

There are further rare *CFI* variants described in aHUS and AMD [D519N, S524V (20)] that result in FI protein with a complete or near complete lack of function which may display a dominant negative effect. In addition to inactive FI variants, FH variants such as R166W may also exhibit a dominant negative effect as a result of almost normal binding to C3b but with an impaired ability to form a TMC impacting C3b cleavage by FI.

The dominant negative effect of inactive FI proteins may have clinical implications since supplementation of FI by sub-retinal gene therapy currently being trialled for the treatment of dry AMD (Gyroscope therapeutics; NCT04566445, NCT04437368) (49) may be less effective in patients with variants like I340T. In these instances, excess FI may need to be added to the eye to counteract the dominant negative effect. It may also be predicted that dominant negative effects in FH variants (e.g. R166W) may similarly impair FH supplementation studies.

In summary, the ability to produce recombinant fully processed FI is a significant technological advance, generating recombinant FI for accurate functional analysis, with the potential for efficient large scale industrial production for use as a biologic, or for incorporation into gene therapy in clinical use.

The method described herein for the building of the complement AP regulatory TMC on a carboxymethyl chip surface and analysis by SPR has the potential to add significant sensitivity and granularity in genetic variant and/or complement drug characterisation for use in clinical trial stratification, diagnostics, drug development and personalised treatment of AMD or other complement -mediated diseases. The dominant negative effect of an inactive FI variant that is secreted could also have implications for therapeutic application and should be taken into consideration when treating patients with AMD and rare *CFI* variants with supplementation therapy.

## Data availability statement

The raw data supporting the conclusions of this article will be made available by the authors, without undue reservation.

## Author contributions

TH & DK wrote the manuscript. DK, KM and AL, conceived and directed the study. TH, TC, KS-J, VB, AB, EW, VS, DS, NT, and CH, generated & purified proteins, undertook complement functional assays, undertook surface plasmon resonance assays and analysed relevant data. All authors contributed to the article and approved the submitted version.
