# Supplementary material for: A novel method for real-time analysis of the complement C3b:FH:FI complex reveals dominant negative CFI variants in age-related macular degeneration
Source: Front Immunol. 2022 Dec 28;13:1028760. doi: 10.3389/fimmu.2022.1028760 (PMC9832388; doi:10.3389/fimmu.2022.1028760)
Supplement: Supplementary file 1 [file DataSheet_1.pdf]

Supplementary Figure 1 – Comparison of enzymatic activity of serum purified FI vs recombinant FI from IRES FI vector

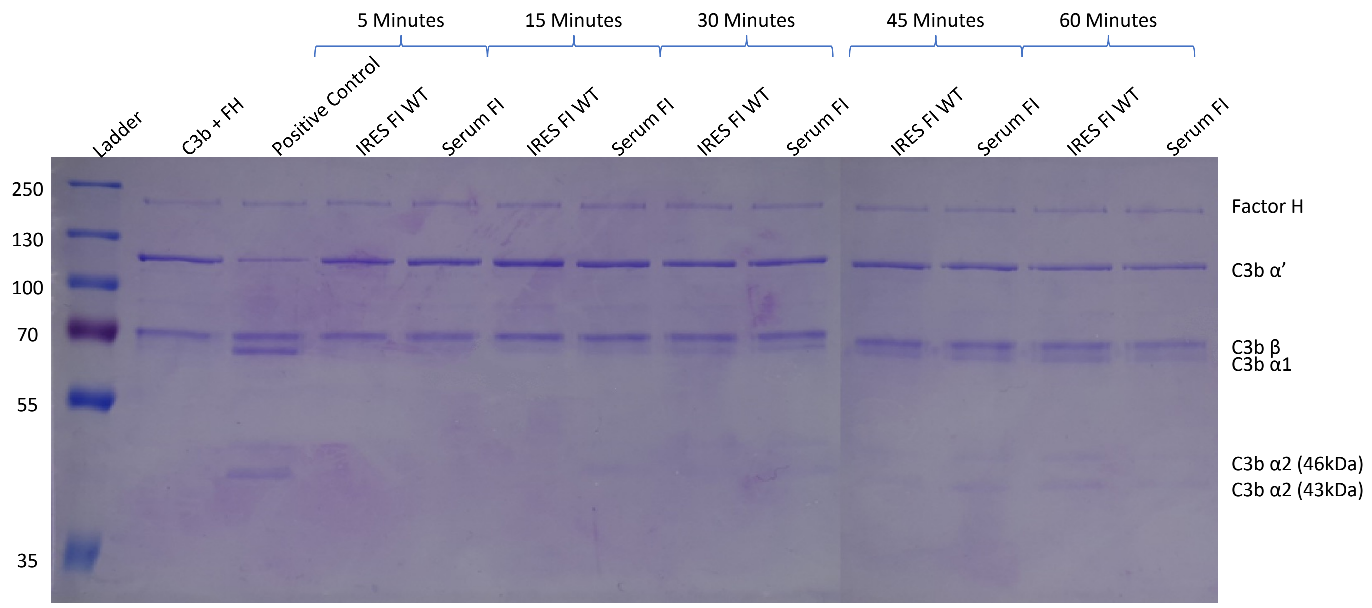

Supplementary Figure 1 – SDS-PAGE visualisation of the fluid phase cofactor (FH) activity of IRES FI compared to serum purified FI, over time. SDS-PAGE showing proteolytic activity of IRES FI and serum purified FI across a range of timepoints (5 – 60 minutes). Activity assessed by the ability of Factor I in combination with its cofactor FH, to cleave C3b to its inactive form iC3b. C3b cleavage was indicated by a reduction in α'-chain density and the appearance of α1 and the two α2 bands. All samples were run under reducing conditions with a PageRuler Prestained Protein ladder (10 – 250 kDa).

Supplementary Figure 2 – S525A FI: a model inactive FI variant

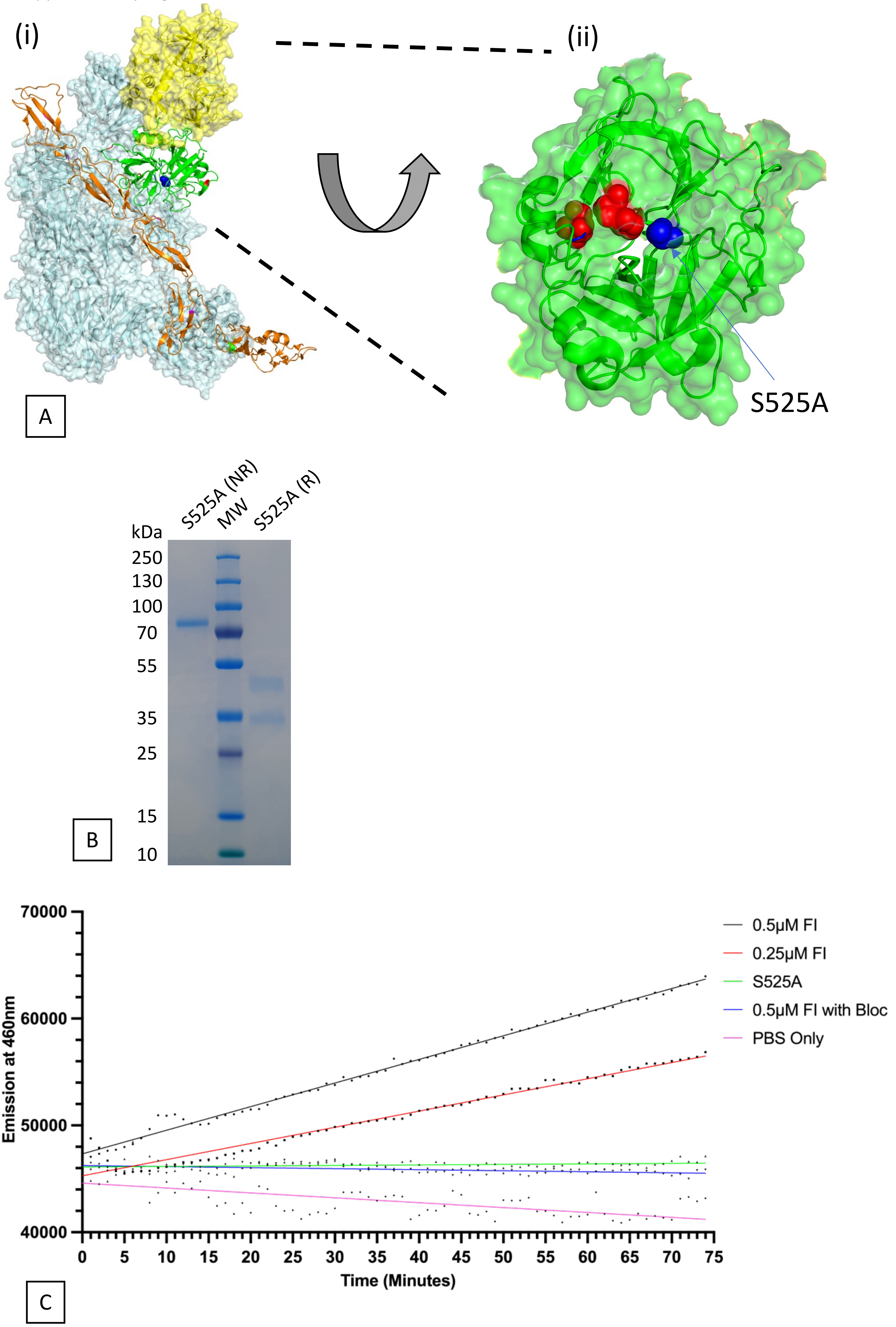

Supplemental Figure 2 - S525A FI: a model inactive FI variant (A) (i) shows the alternative pathway regulatory tri-molecular complex (TMC) comprising C3b, FH1-4, and FI. The FI light chain is green and transparent demonstrating the underlying cartoon structure, with the heavy chain displayed in yellow. C3b is in pale cyan and FH CCPs 1-4 and 19-20 are displayed by orange cartoon (ii) demonstrates a higher magnification of complement factor I with one of the amino acids of factor I altered (S525A, blue spheres within a solvent exposed cavity) to produce a factor I backbone that will bind to the TMC but not cleave C3b. H380 and D429 of the catalytic triad are highlighted by a red spheres.

(B) SDS-PAGE of inactive FI (S525A) generated with the IRES vector stained with Coomassie blue. SDS-PAGE of reduced and non-reduced inactive FI stained with Coomassie Blue.

(C) Comparison of Boc-Asp(OBzl)-Pro-Arg-AMC cleavage by active and inactive FI. Activity of FI in the absence of cofactor as determined by the release of fluorogenic AMC substrate. Emission at 460nm is plotted against time. Bloc = Pefabloc SC (Sigma, UK).

Supplementary Figure 3 – SDS-PAGE for purified complement proteins

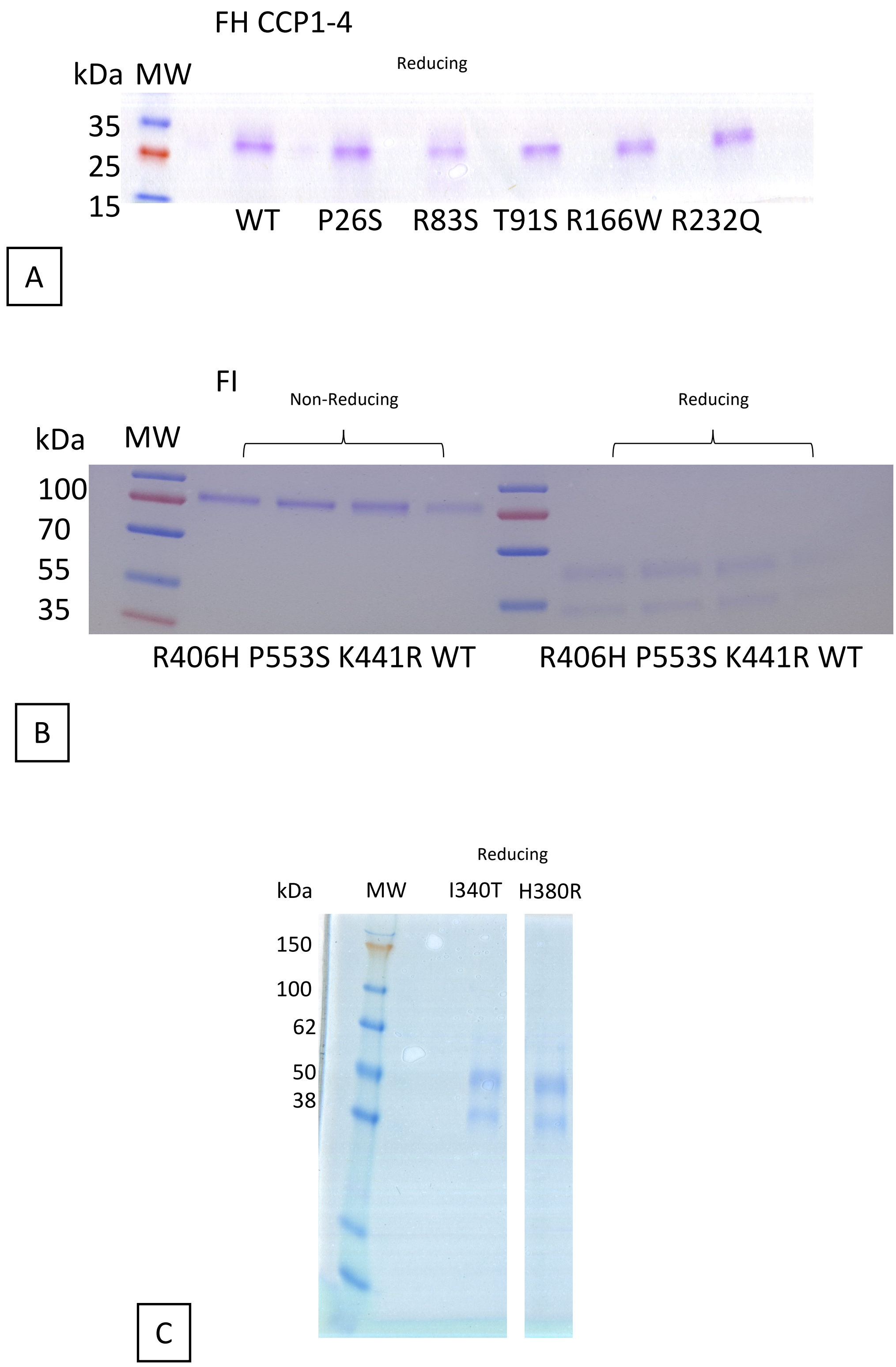

Supplemental Figure 3 - SDS-PAGE for purified complement proteins. Standard reducing SDS-PAGE followed by staining with Coomassie blue revealed fully folded, pure (A) FH CCP1-4 WT, P26S, R83S, T91S, R166W, R232Q (500ng); (B) FI WT, R406H, K441R, P553S (500ng); (C) FI I340T, H380R (500ng); Samples were reduced using 10% betamecaptoethanol.

Supplementary Figure 4 – AP Regulatory TMC Building with Active FI at 4°C

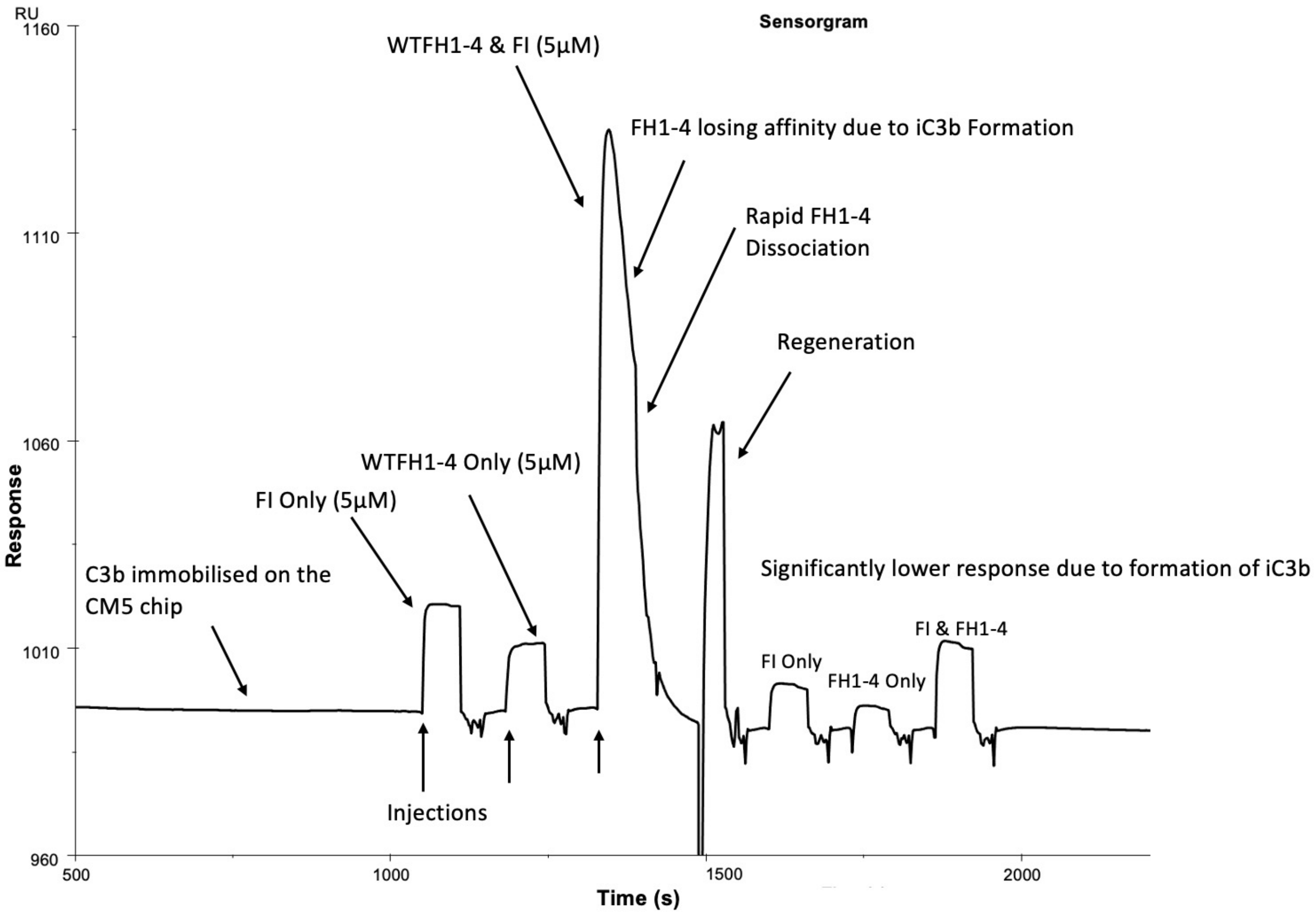

Supplementary Figure 4 - AP Regulatory TMC Building with Active FI at 4°C. Using the BIAcore S200, 5µM of WT active FI, followed by 5µM WT FH1-4, followed by a mix of the two (both at 5µM), were injected onto a C3b coupled chip surface using the BIAcore S200 in running buffer (HBS + 0.05% Tween 20). Thermal slowing of the kinetics of the reaction failed to prevent cleavage of C3b hindering interrogation of AP regulatory TMC formation. Subsequent injections after regeneration (with pH4 10mM sodium acetate, 1M NaCl) resulted in substantially lower binding of each protein individually and combined.

Supplementary Figure 5 – FH1-4 and inactive (S525A) FI 125nM injections onto a C3b coupled surface

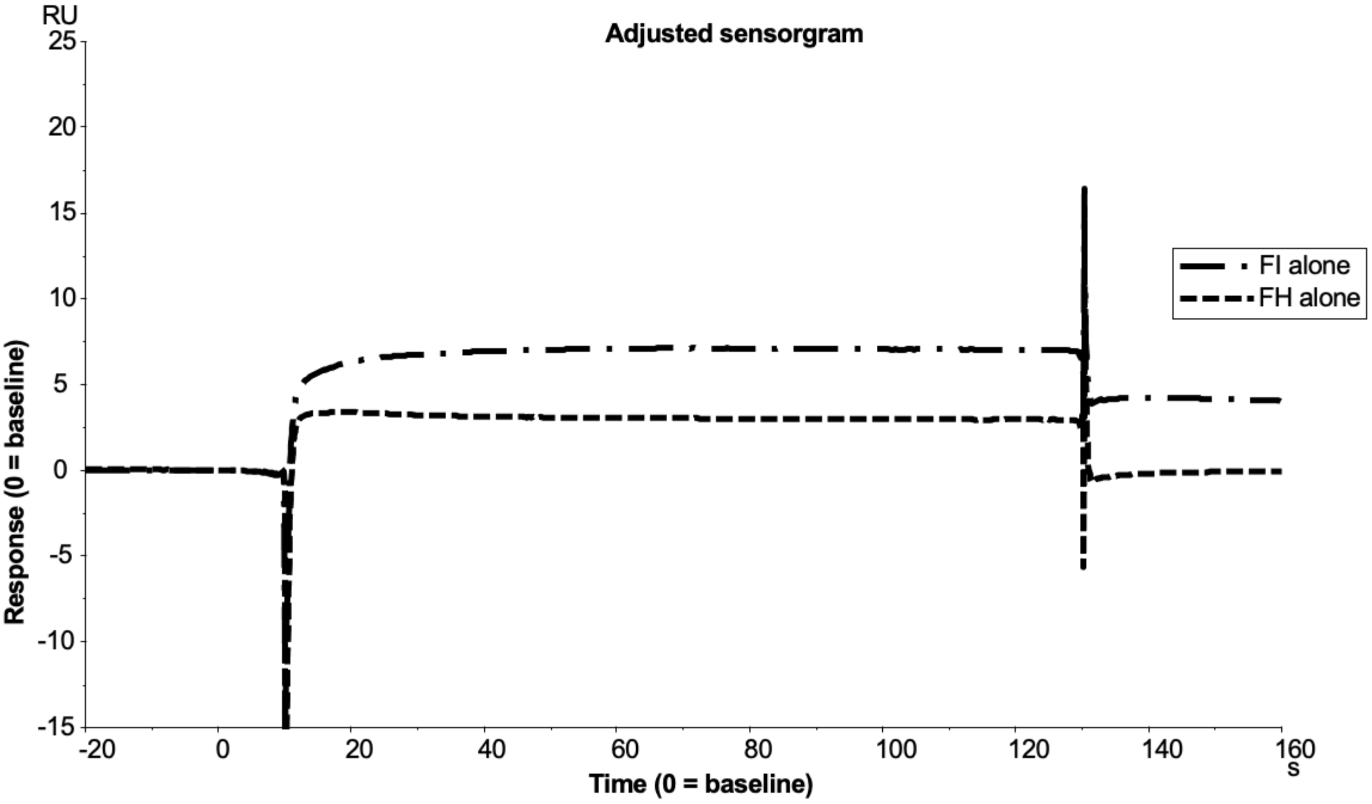

Supplementary Figure 5 – FH1-4 and inactive (S525A) FI 118nM injections onto a C3b –coupled surface. FI and FH were injected, at 118nM, onto an 1000RU thiol coupled CM5 chip surface individually using the BIAcore S200 to check for steady state binding of both proteins to C3b. Only a ~5RU response was observed.

Supplementary Figure 6 – Additional FH1-4 variant TMC building on an amine coupled chip surface.

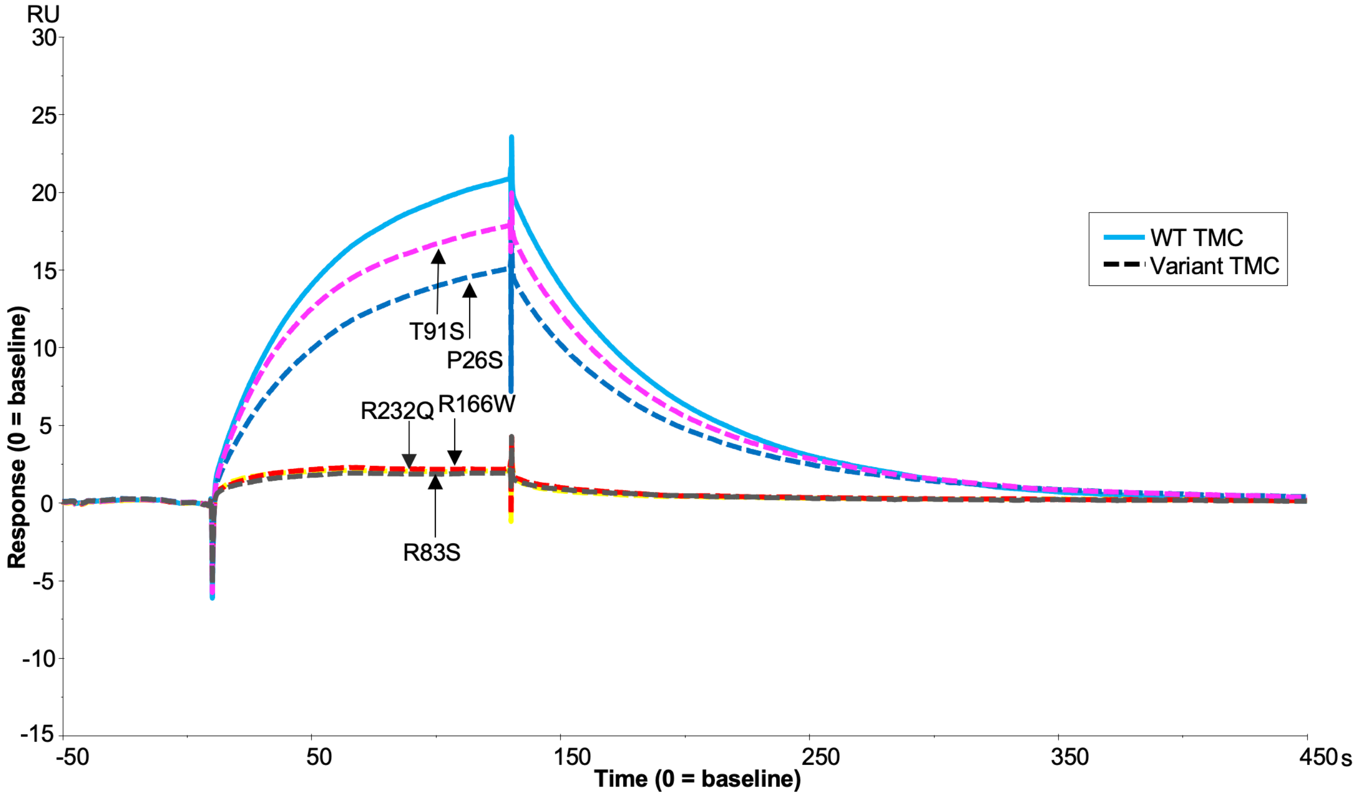

Supplementary Figure 6 – FH1-4 variant TMC building on an amine coupled chip surface. In PBST, FI and FH1-4 variants were injected simultaneously onto an 800RU C3b amine coupled chip surface at 59nM. The displayed sensorgrams are normalised to a blank flow cell and is representative of at least 2 experiments at multiple concentrations of FH1-4.

Supplementary Figure 7 – AP C3 convertase building before and after inactive and active FI TMC building: thiol and amine coupled

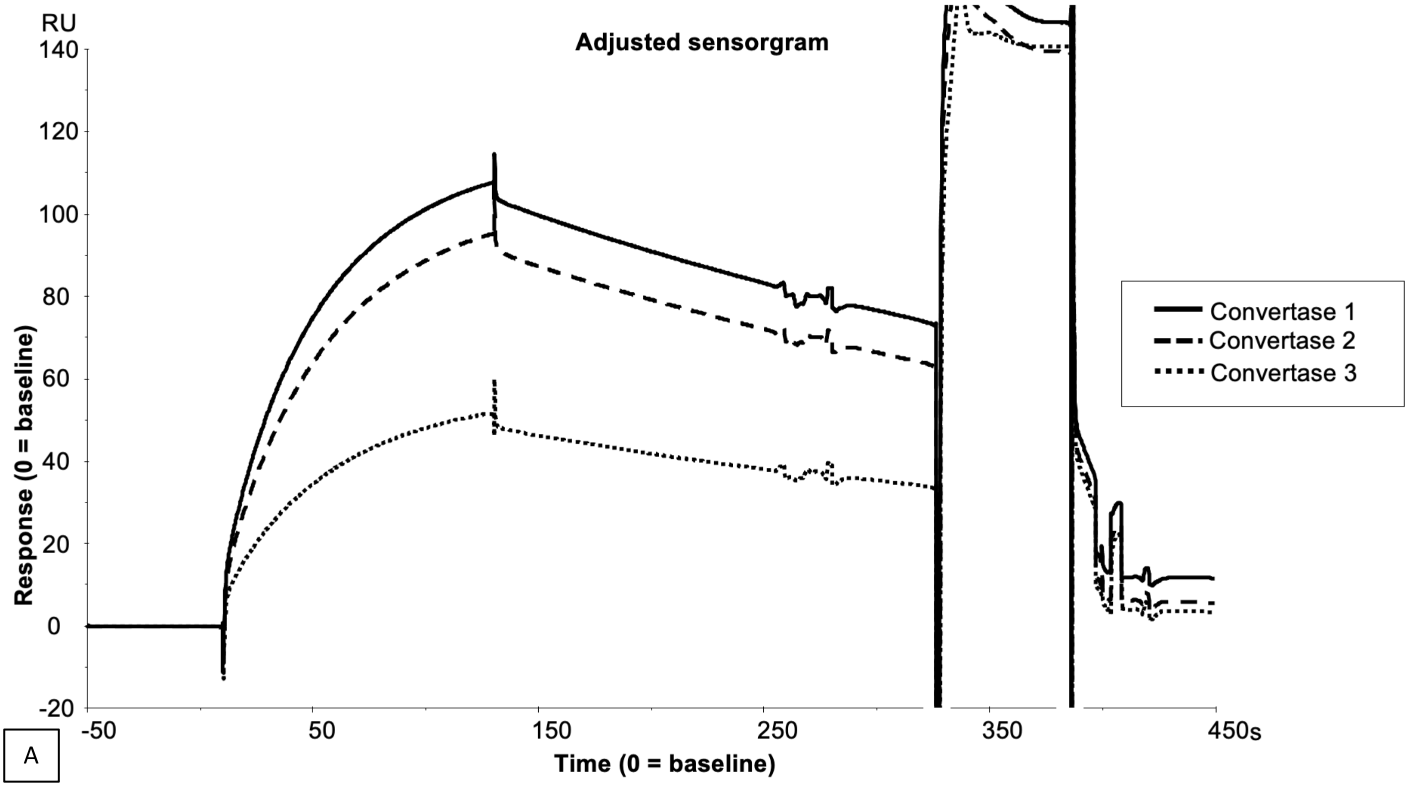

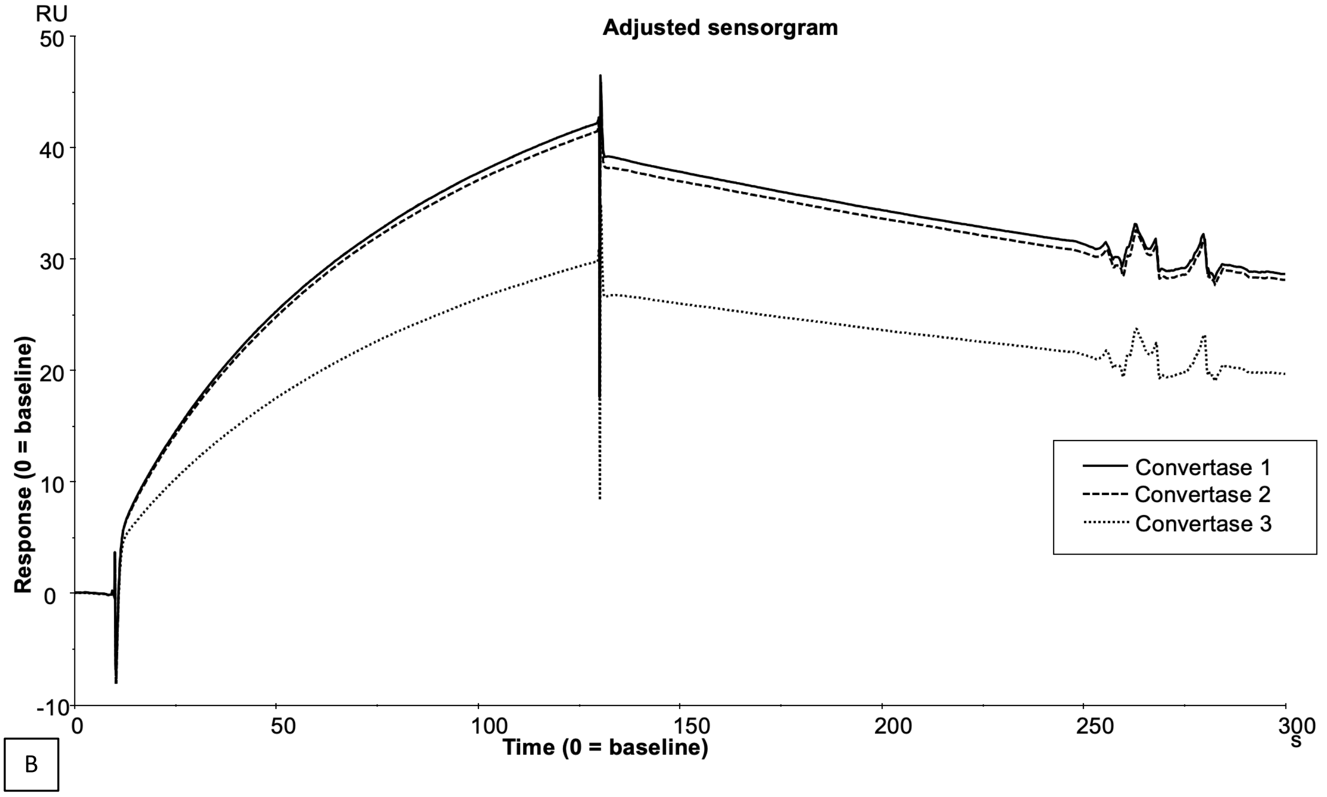

Supplementary Figure 7 – AP C3 convertase building before and after inactive and active FI TMC building: thiol coupled and amine coupled.

Using the BIAcore S200 in PBST, to test for degradation of the chip surface before and after the injections of FH1-4 variants with inactive FI for comparative analysis, the C3 convertase (C3bBb) was built on the C3b (800-10000RU) coupled chip by injection of 500nM FB and 60nM FD for 90 seconds at 20µL/min with a 90 second dissociation time (convertase 1 and convertase 2).

As an additional and final test, active WT FI and FH1-4 were then flowed across (at 125nM) to cleave C3b and following this, FD and FB were added at standard convertase building concentrations revealing a lower RU increase (convertase 3) consistent with C3b cleavage.

(A) The thiol coupled chip surface displayed a loss of activity over time and after cleavage by WT active FI.

(B) The amine coupled chip surface displayed loss of activity only after cleavage by WT active FI.

Supplementary Figure 8 – Additional FI variant analyses. (A) TMC building on amine coupled chip

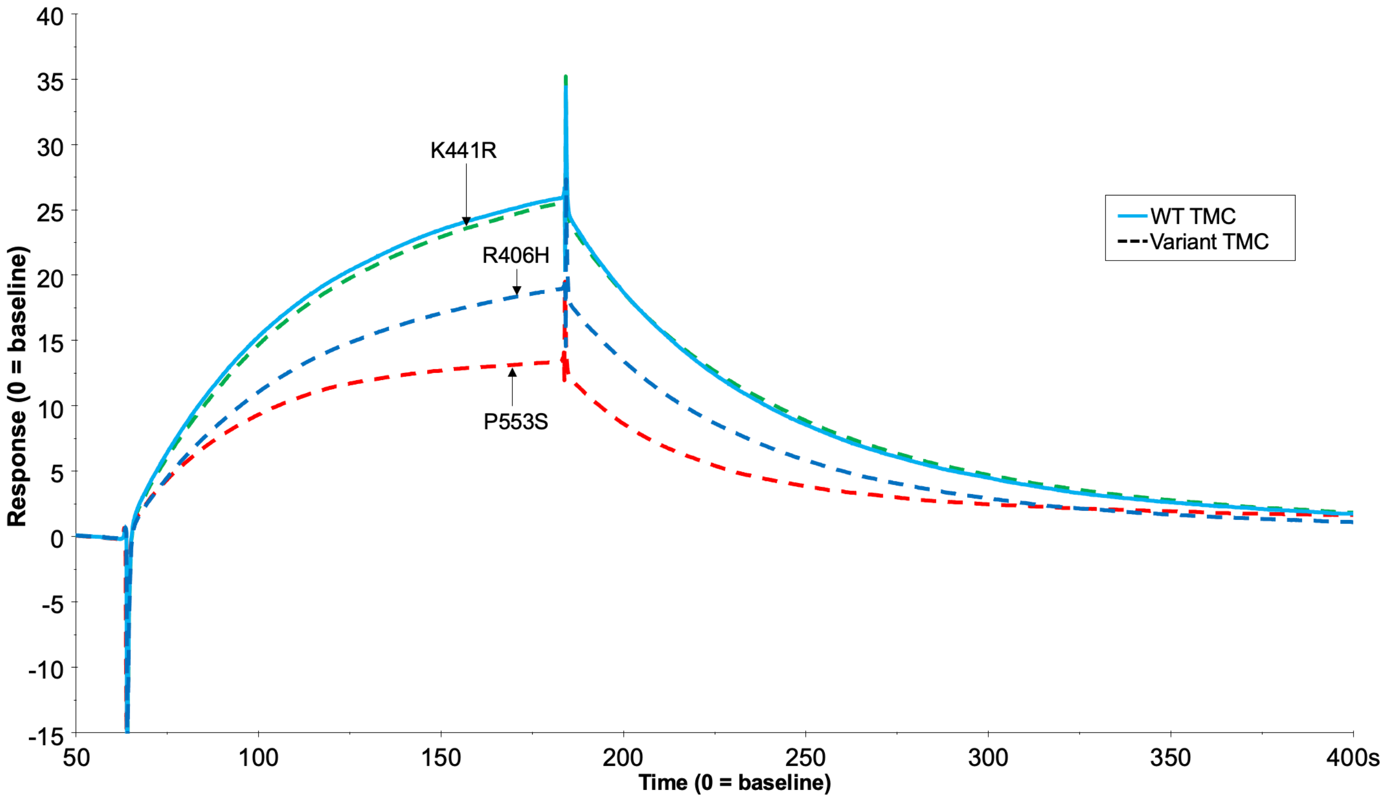

Supplementary Figure 8 – FI variant TMC building on an amine coupled chip. Using the BIAcore S200 in PBST, FH1-4 and FI variants were injected simultaneously onto an 1000RU C3b amine coupled chip surface at 62.5nM. The displayed sensorgrams are normalised to a blank flow cell and is representative of at least 2 experiments at multiple concentrations of FI.

Supplementary Figure 9 – AP regulatory TMC building with active and inactive FI: amine coupled

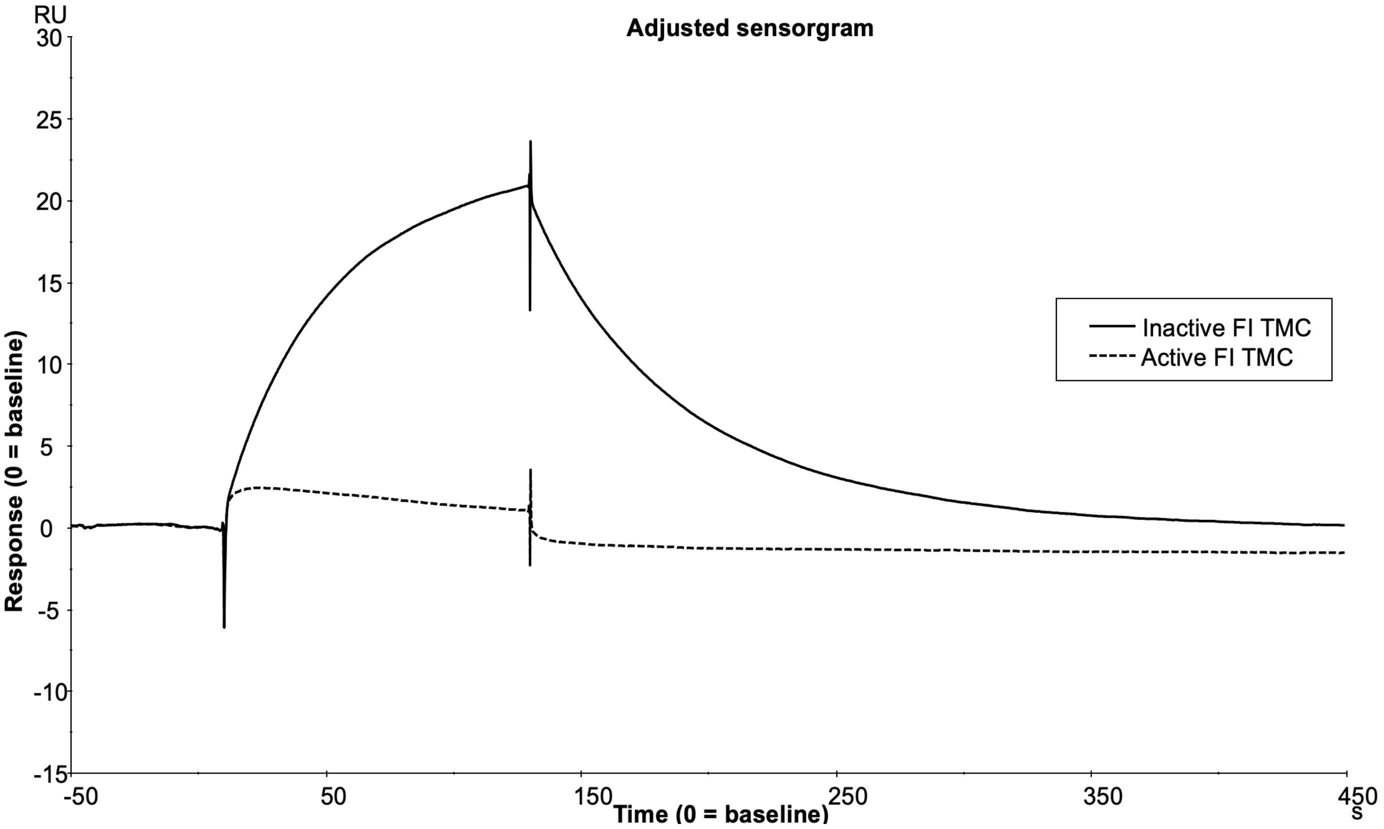

Supplementary Figure 9 – AP regulatory TMC building with active and inactive FI: amine coupled. FI (active, dashed line; inactive (S525A), solid line) and FH1-4 were injected at 125nM onto an 800RU C3b amine coupled CM5 chip surface using the Biacore S200.

Supplementary Figure 10 – H380R FI dominant negative effect

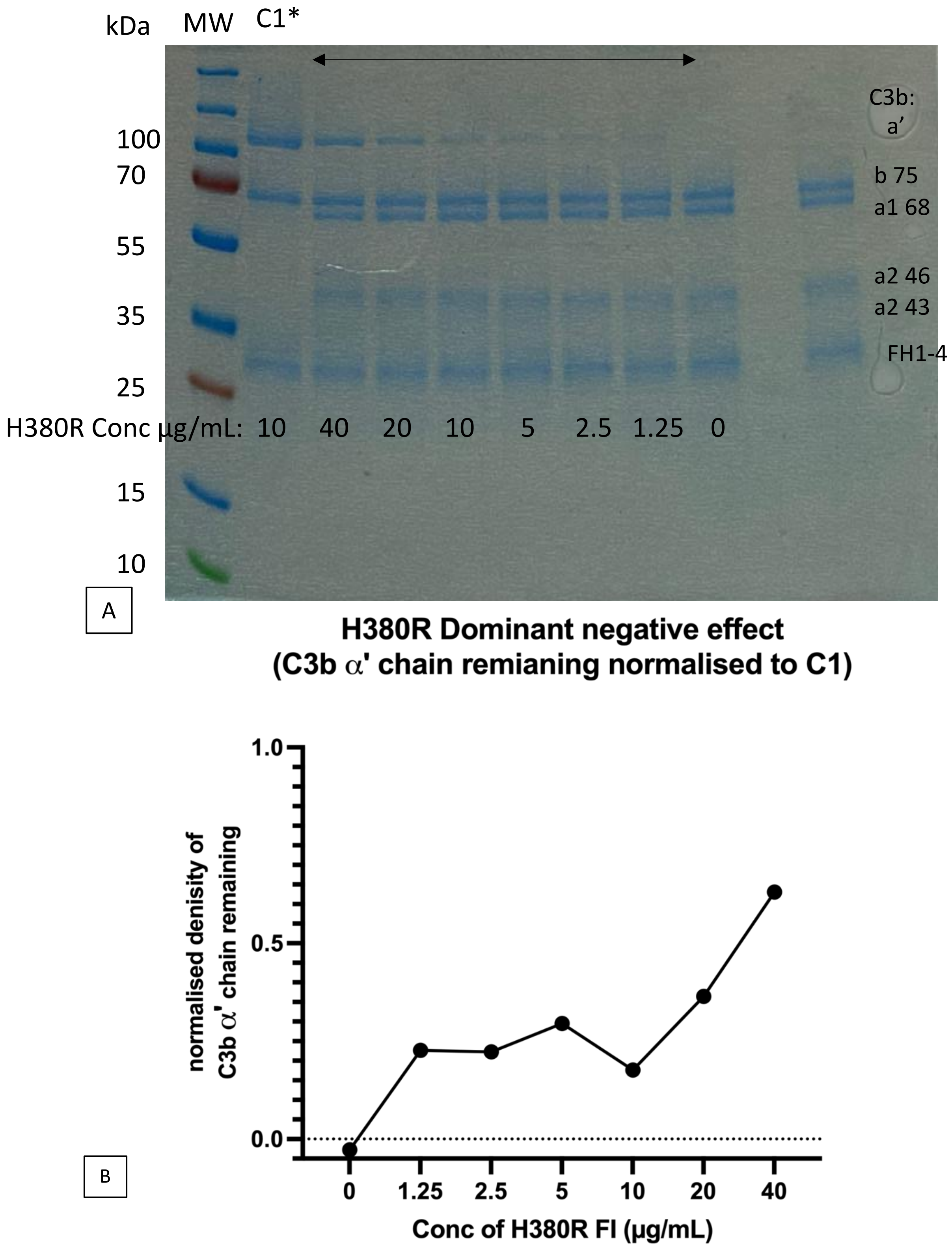

Supplementary Figure 10 – H380R FI dominant negative effect. (A) A fluid phase cofactor assay revealed a dominant negative effect of inactive (H380R) FI on active WT FI. Addition of 7.5µL of 0, 1.25, 2.5, 5, 10, 20 and 40 µg/mL inactive FI to a standard cofactor assay (1µg C3b, 75ng WT FI and 500ng FH1-4), before incubation for 60 mins, resulted in an inhibitory effect as shown by lower levels of breakdown of the C3b α'-chain when 40 µg/mL H380R FI was spiked compared to a 0 µg/mL control lane. *C1 = control 1 (negative control including C3b, FH1-4 and H380R FI but no WT FI). (B) Densitometry analysis. Licor ImageStudio™ Lite with median background subtraction was utilised to perform densitometry analysis on the SDS-PAGE gel resulting from the H380R dominant negative fluid phase assay. Plotted are densities of C3b α'-chain remaining normalised to the β-chain for each test and normalised to the negative control (C1) value. Graph produced using GraphPad Prism V8.

Supplementary Figure 11 – Human serum albumin fluid phase FI activity control – dominant negative effect is not due to non-specific protein interference.

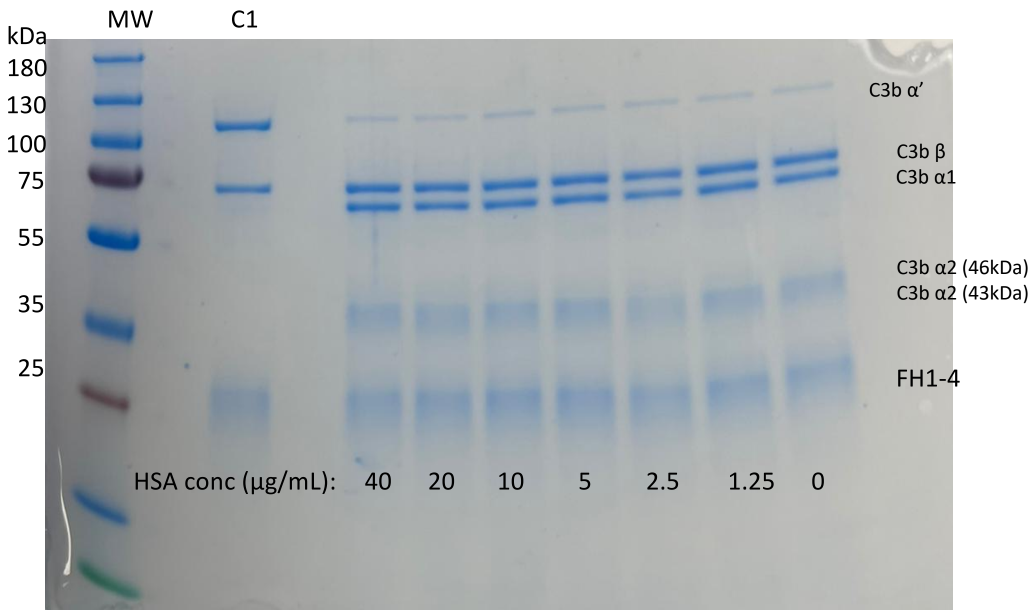

A

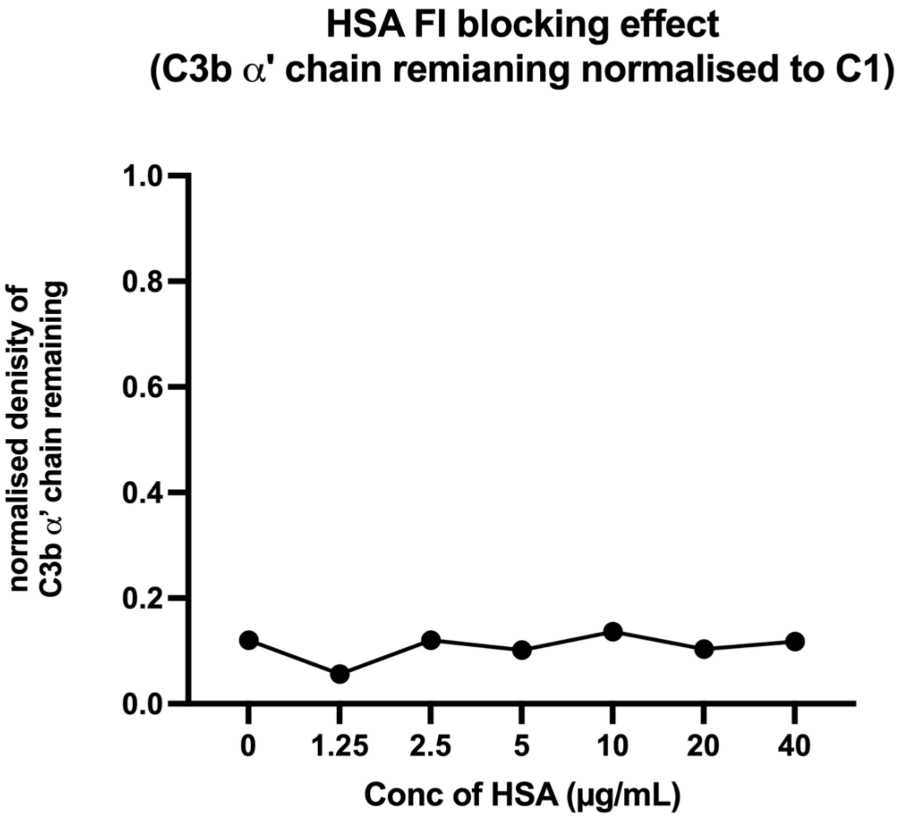

B

Supplementary Figure 11 – Human serum albumin fluid phase FI activity control. (A) Fluid phase assay SDS-PAGE. Addition of 7.5µL of 0, 1.25, 2.5, 5, 10, 20 and 40 µg/mL human serum albumin (HSA, Sigma, UK) to a standard cofactor assay (1µg C3b, 75ng WT FI and 500ng FH1-4), before incubation for 60 mins, resulted in no inhibitory effect. C1 = control 1 (negative control including C3b, FH1-4 and no WT FI).
(B) Densitometry analysis. Licor ImageStudio Lite with median background subtraction was utilised to perform densitometry analysis on the SDS-PAGE gel resulting from the HSA supplemented fluid phase assay displayed in (A). Plotted are densities of C3b α’ -chain remaining normalised to the beta chain for each test and normalised to the negative control (C1) value. Graph produced using GraphPad Prism V8.

Supplemental figure 12 - SPR analysis of the H380R FI dominant negative effect.

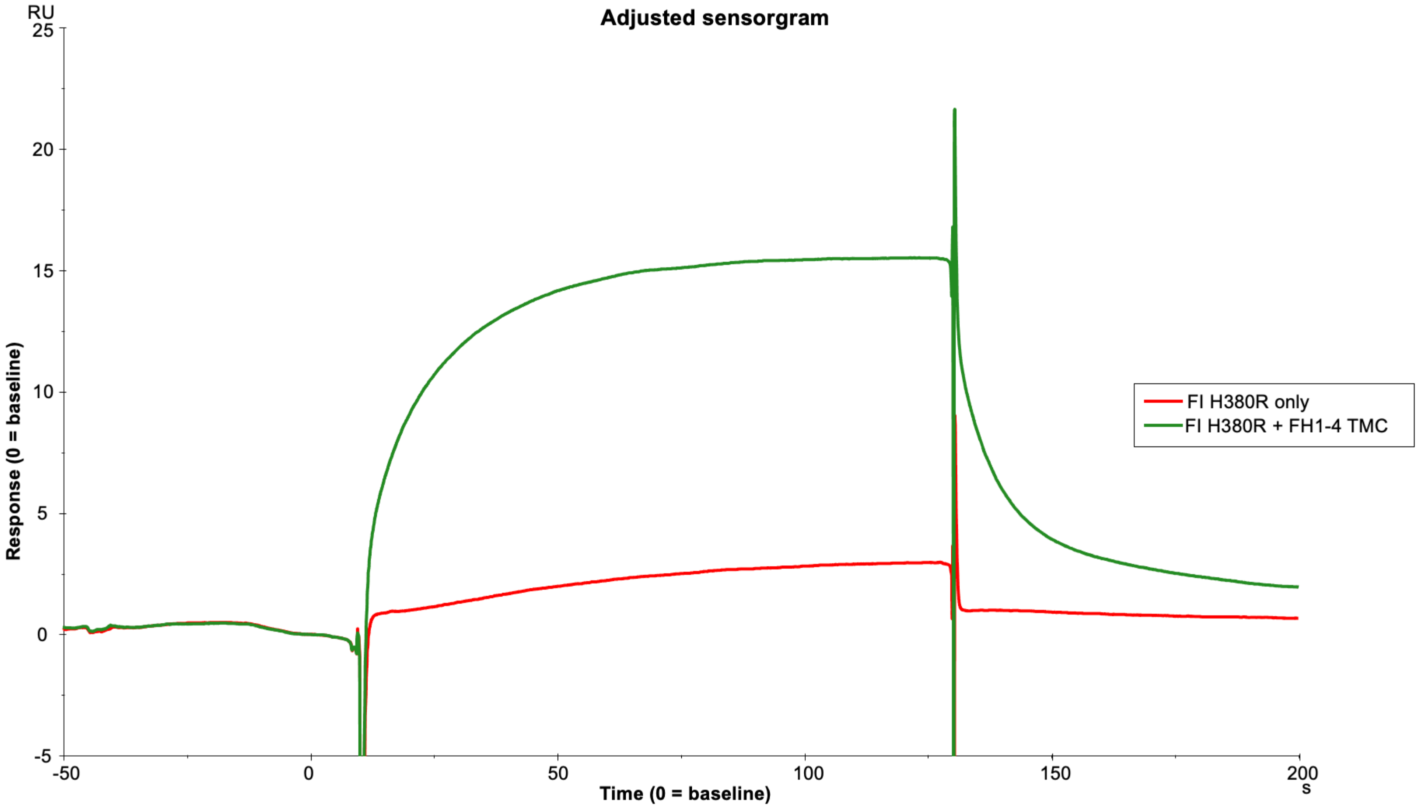

Supplemental figure 12 - SPR analysis of the H380R FI dominant negative effect. Injection of 125nM of H380R FI with 125nM of FH1-4 onto a C3b amine coupled (~1000RU) CM5 chip using the BIAcore S200 resulted in the building of an AP regulatory TMC (of ~15RU) (green line) which did not immediately dissociate when the injection finished after 120 seconds. Meanwhile, injection of 125nM of H380R FI alone onto the same surface resulted in only a ~2RU response. SPR experiments are indicative of at least 2 repeats.

Supplementary Figure 13 – I340T C3b interaction in absence of FH

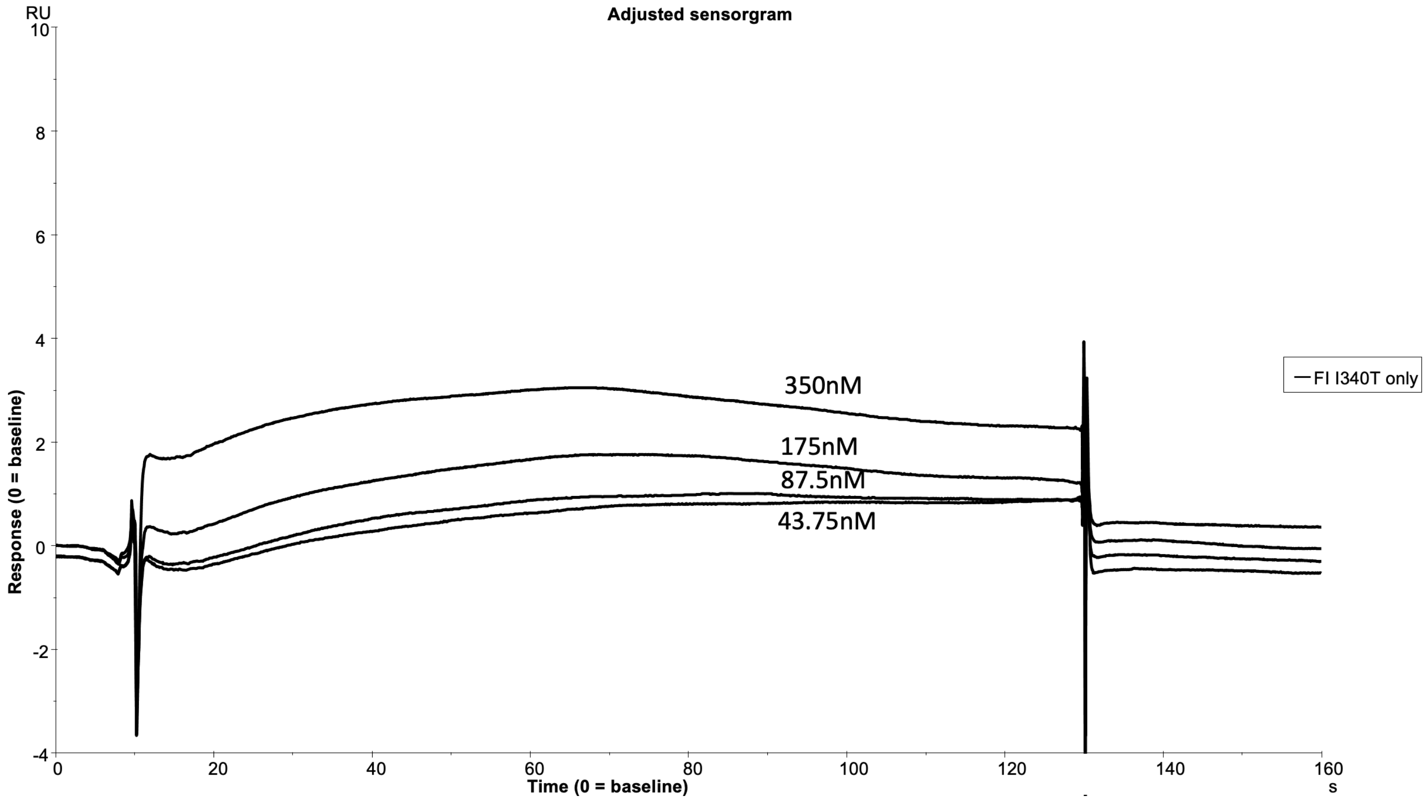

Supplementary Figure 13 – I340T C3b interaction in absence of FH. (A) Using the BIAcore S200 in PBST, I340T FI was injected in a 1:2 serial dilution starting at 350nM onto a 1000RU C3b coupled CM5 chip surface to show that the protein has affinity for C3b in the absence of FH CCP 1-4.

Supplementary Figure 14 – AP regulatory TMC building with soluble CR1 and FHL-1

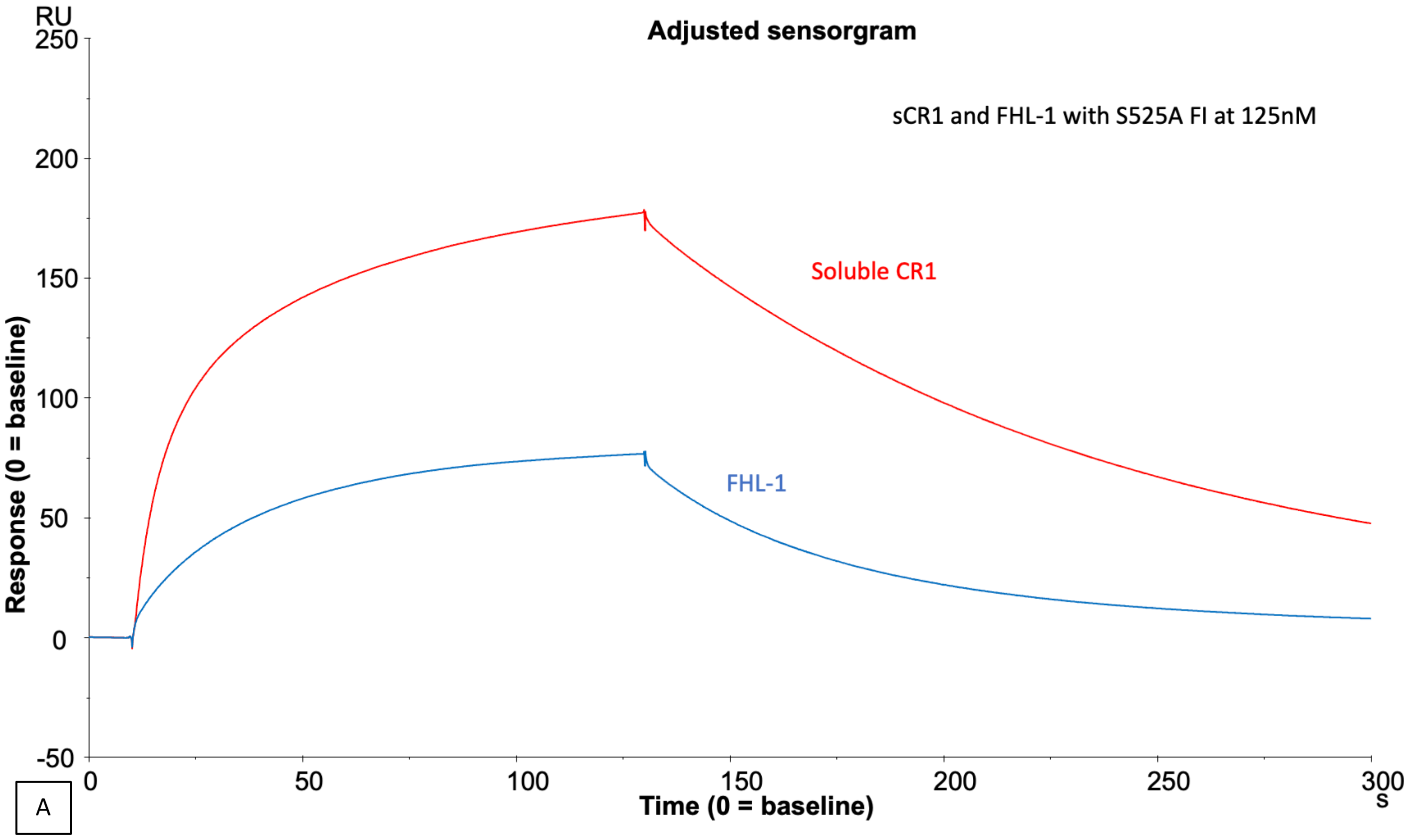

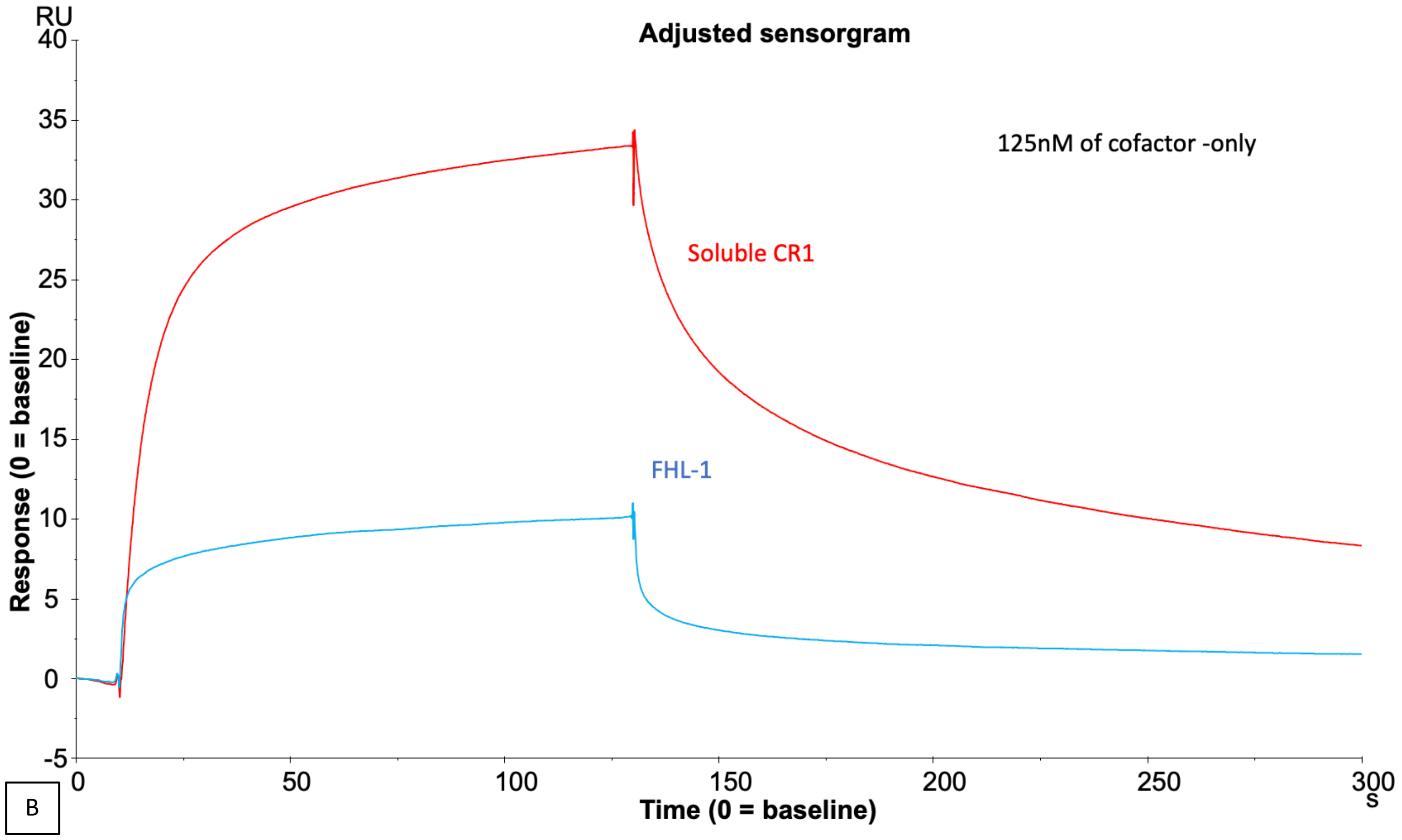

Supplementary Figure 14 – AP regulatory TMC building with soluble CR1 and FHL-1. (A) Soluble CR1 (A gift to the lab from Paul Morgan, Cardiff University) (red line) and FHL-1 (in-house produced using Pichia) (blue line) were injected at 125nM with 125nM of inactive FI onto a 1000RU C3b thiol coupled CM5 chip surface. (B) 125nM soluble CR1 (red line) or FHL-1 (blue line) alone were injected onto the same C3b thiol coupled surface.

Supplementary Figure 15 – AP regulatory TMC building with soluble MCP

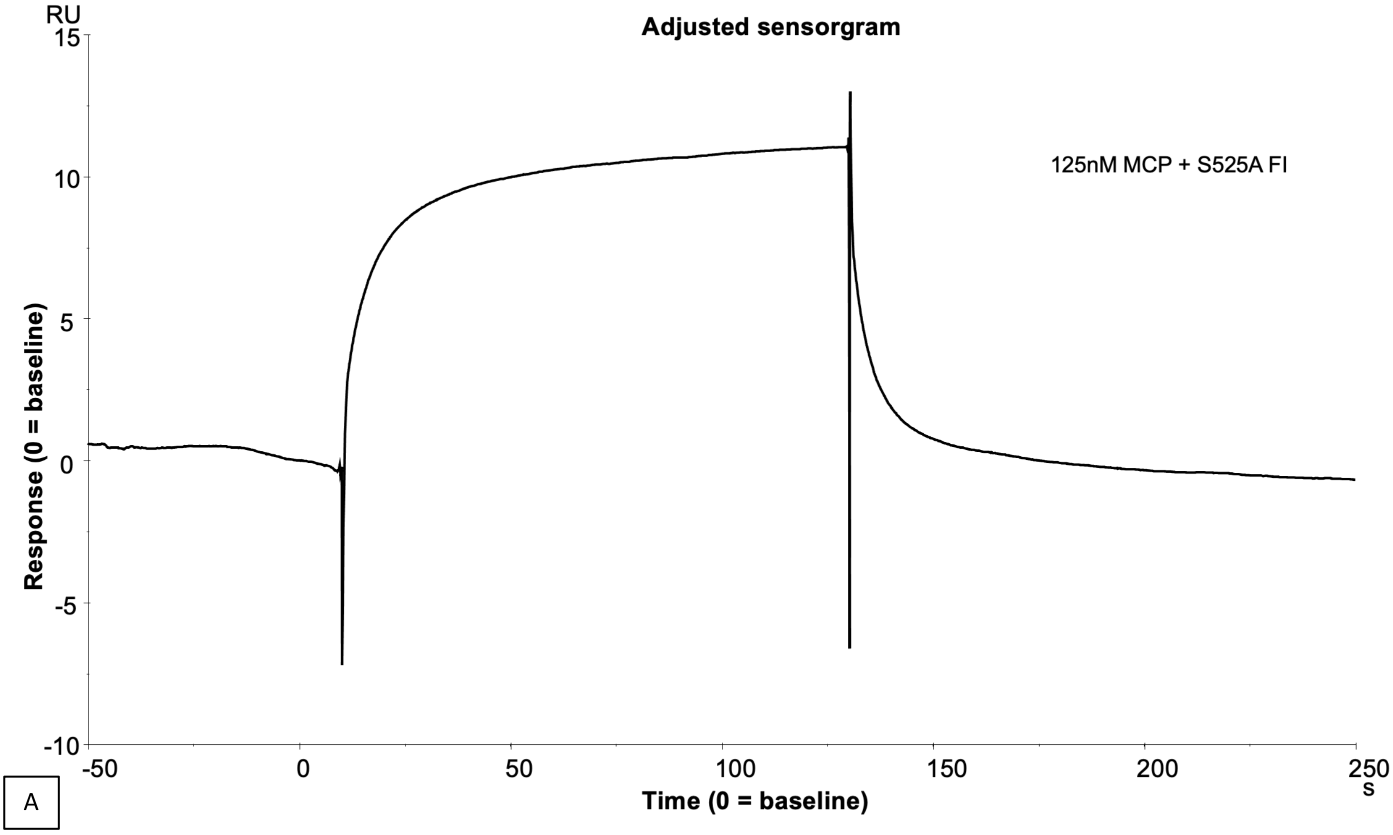

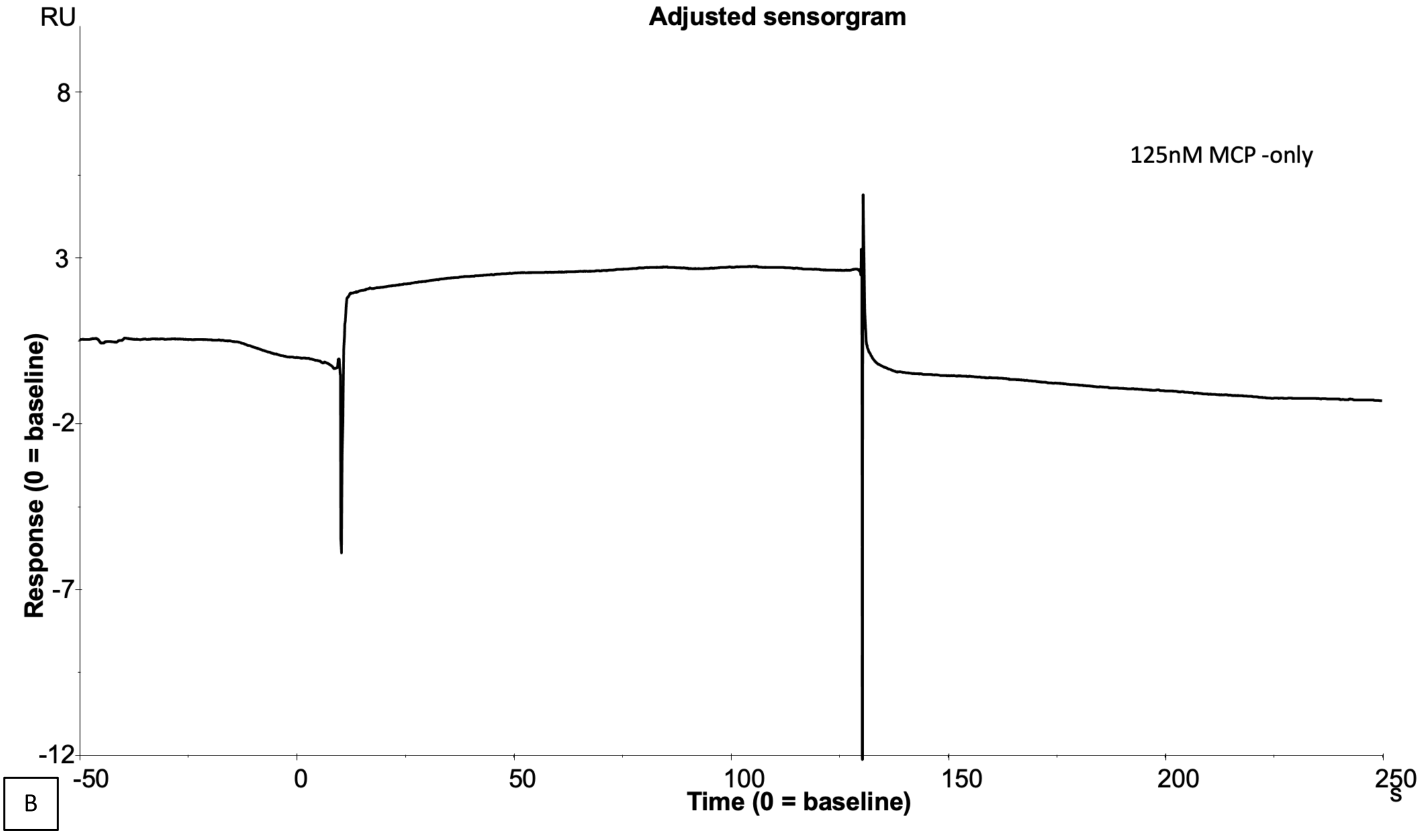

Supplementary Figure 15 – AP regulatory TMC building with soluble MCP. (A) Soluble MCP (A gift to Claire Harris from Susan Lea, Oxford University) (black line) was injected at 125nM with 125nM of inactive FI onto a 1000RU C3b thiol coupled CM5 chip surface using the BIAcore S200. (B) 125nM soluble MCP alone was injected onto the same C3b thiol coupled surface.

Supplementary References

- X. Xue *et al.*, Regulator-dependent mechanisms of C3b processing by factor I allow differentiation of immune responses. *Nat Struct Mol Biol* **24**, 643-651 (2017).
