# Supplementary material for: A novel method for real-time analysis of the complement C3b:FH:FI complex reveals dominant negative CFI variants in age-related macular degeneration
Source: Front Immunol. 2022 Dec 28;13:1028760. doi: 10.3389/fimmu.2022.1028760 (PMC9832388; doi:10.3389/fimmu.2022.1028760)
Supplement: Supplementary file 2 [file Table_1.docx]

Supplementary Table 1: *CFI* variant SDM and sequencing primers.

| **Mutation (in *CFI*)** | **Base 1** | **Base 2** | **Primer (forward)** | **Primer (reverse)** |
| --- | --- | --- | --- | --- |
| R406H  Mutagenesis Primer | C**G**T | C**A**T | 5’-ggatacaccccgaccttaaacatatagtaattgaatacgtg-3’ | 5’- cacgtattcaattactatatgtttaaggtcggggtgtatcc-3’ |
| K441R  Mutagenesis Primer | A**A**A | A**G**A | 5’- tgaaatgaaaaaagacggaaacagaaaagattgtgagctgc-3’ | 5’- gcagctcacaatcttttctgtttccgtcttttttcatttca-3’ |
| P553S  Mutagenesis Primer | **C**CA | **T**CA | 5’- ggaaaactgtggaaaatcagagttcccaggtgtttacaccaaagtggc-3’ | 5’- gccactttggtgtaaacacctgggaactctgattttccacagttttcc-3’ |
| S525A  Mutagenesis Primer | **T**CT | **G**TC | 5’- cctgtaaaggggacgctggaggccccttagtctgtatggatgc-3’ | 5’- gcatccatacagactaaggggcctccagcgtcccctttacagg-3’ |
| *CFI* Sequencing Primer 1 | N/A | N/A | 5’-ccacttaaggttttgcaaggtc-3’ | N/A |
| *CFI* Sequencing Primer 2 | N/A | N/A | 5’-agtgtttccttgaagcatgg-3’ | N/A |
| *CFI* Sequencing Primer 3 | N/A | N/A | 5’-cagatgaaagcctgtgatgg-3’ | N/A |
| *CFI* Sequencing Primer 4 | N/A | N/A | 5’-atgccagtggaatcacctgt-3’ | N/A |

Supplementary Table 2: *CFH*1-4 variant SDM and sequencing primers.

| **Mutation (in *CFH*1-4)** | **Base 1** | **Base 2** | **Primer (forward)** | **Primer (reverse)** |
| --- | --- | --- | --- | --- |
| P26S Mutagenesis Primer | **C**CA | **T**CA | 5'-cagaagattgcaatgaacttccttcaagaagaaatacagaaattctg-3' | 5'-cagaatttctgtatttcttcttgaaggaagttcattgcaatcttctg-3' |
| T91S Mutagenesis Primer | **A**CT | **T**CT | 5'-cctgtggacatcctggagattctccttttggtact-3' | 5'-agtaccaaaaggagaatctccaggatgtccacagg-3' |
| R166W Mutagenesis Primer | **C**GG | **T**GG | 5'-cagtagtgcaatggaaccagattgggaataccattttgg-3' | 5'-ccaaaatggtattcccaatctggttccattgcactactg-3' |
| R232Q Mutagenesis Primer | C**G**A | C**A**A | 5'-tcagaagattatttataaggagaatgaacaatttcaatataaatgtaacatgggttatg-3' | 5'-cataacccatgttacatttatattgaaattgttcattctccttataaataatcttctga-3' |
| pPICZαB Sequencing Primer | N/A | N/A | 5'-cgggttattgtttataaatac-3' | 5'-gtcgacggcgctattcagatc-3' |

Supplementary Table 3 – *CFI* Variant Details

| **cDNA bp change (NM_000204.4)** | **Factor I Amino Acid Change and CADD Score** | **Domain** | **Reference(s)** | **Known Conditions** | **In Vitro / Recombinant Analysis / Other remarks** | **In Vivo Analysis** |
| --- | --- | --- | --- | --- | --- | --- |
| 1019T>C | p.Ile340Thr  (CADD 23.6) | Serine Protease | Geelen et al. 2007; Le Quintrec et al. 2008; Kavanagh et al. 2008; Westra et al. 2010; Geerdink et al. 2012; Seddon et al. 2013; Bresin et al. 2013; Haerynck et al. 2013; Kavanagh et al. 2015; Fritsche et al. 2016; Geerlings et al. 2018; Osborne et al. 2018; El Sissy et al. 2019; Altmann et al. 2020 | aHUS; AMD; C3G/MPGN; cerebral inflammation; FI or complement deficiency | Normal secretion, almost completely impaired fluid phase C3b and C4b degradation activity (Kavanagh et al. 2008) | One heterozygous patient had a normal level of FI (Geelen et al. 2007), One CrGN patient with a normal FI level (Le Quintrec et al. 2008), One patient with complete FI deficiency causing recurrent Aseptic Meningo-Encephalitis in heterozygousity with Asp524Val had a normal FI level (Haernyck et al. 2012), One heterozygous patient with aHUS had a normal level of FI (Bresin et al. 2013), 3 aHUS heterozygous patients with normal levels (Osborne et al. 2018); One patient with fulminant cerebral inflammation had normal FI levels in compound heterozygousity with Asp519Asn (Altmann et al. 2020); One patient with complete clinical FI deficiency, a normal FI level and no AP activity, in compound heterozygousity with Asp524Val (Naesen et al. 2020) |
| 1139A>G | p.His380Arg  (CADD 27.1) | Serine Protease | Franco-Jarava et al. 2017 | FI Deficiency | H380 is the histidine of the catalytic triad of the FI serine protease domain | ~50% reduction of FI in the serum of a homozygous carrier with clinical FI deficiency with no detectable AP activity, 2 heterozygous family members with normal levels and 1 with a slightly reduced level, a second unaffected homozygous family member had low FI levels (Franco-Jarava et al. 2017) |
| 1217G>A | p.Arg406His  (CADD 7.295) | Serine Protease | Kavanagh et al. 2008; Seddon et al. 2013; Kavanagh et al. 2015; Tan et al. 2017; Geerlings et al. 2018; Java et al. 2019 | aHUS; AMD | Normal secretion and functional fluid phase co-factor activity (Kavanagh et al. 2008); Benign in Zebrafish retinal hyaloid vessel formation assay (Tan et al. 2017); Reduced C3b degradation with CR1 and FH as cofactors (Java et al. 2019) | Three heterozygous carriers with aHUS, 2 of which had normal FI levels (Kavanagh 2008) Three heterozygous carriers had normal serum FI levels (2 non-AAMD, 1 AAMD) (Kavanagh et al. 2015); one heterozygous aHUS patient had a normal FI level (Java et al. 2019) |
| 1322A>G | p.Lys441Arg  (CADD 0.002) | Serine Protease | Cayci et al. 2012; Seddon et al. 2013; Bresin et al. 2013; Kavanagh et al. 2015; Fritsche et al. 2016; Tan et al. 2017; Geerlings et al. 2018; Osborne et al. 2018; Shoshany et al. 2019 | aHUS; MPGN/C3G; AMD; | Benign in Zebrafish retinal hyaloid vessel formation assay (Tan et al. 2017) | Normal serum levels in 16 of 18 carriers (11% low) (Kavanagh et al. 2015) |
| 1657C>T | p.Pro553Ser  (CADD 15.35) | Serine Protease | Fang et al. 2008; Bienaime et al. 2010; Seddon et al. 2013; Bresin et al. 2013; Fremeaux-Bacchi et al. 2013; Kavanagh et al. 2015; Geerlings et al. 2017; Tan et al. 2017; Geerlings et al. 2018; Osborne et al. 2018; Java et al. 2019 | aHUS; MPGN/C3G; AMD | Impaired ability to degrade C3b by serum (Geerlings et al. 2017); Benign in Zebrafish retinal hyaloid vessel formation assay (Tan et al. 2017); Normal secretion and function in fluid phase C3b degradation with CR1, MCP and FH (Java et al. 2019) | One heterozygous aHUS patient had a normal FI serum level (Bienaime et al. 2010); 9 carriers in heterozygousity had normal serum levels (8 AAMD and 1 non-AAMD) (Kavanagh et al. 2015); 12 heterozygous carriers had on average the same FI levels as non-carriers (Geerlings et al. 2017); One heterozygous aHUS patient had a normal level of FI (Java et al. 2019) |

Supplementary Table 4 – *CFH* Variant Details

| **cDNA bp change (NM_000186.3)** | **Factor H Amino Acid Change** | **CCP Domain** | **RS number** | **Reference(s)** | **Functional Data** | **CADD Score** | **Polyphen 2** |
| --- | --- | --- | --- | --- | --- | --- | --- |
| 76C>T | p.Pro26Ser | 1 | NA | Triebwasser et al. 2015 | N/D | 25.5 | possibly damaging |
| 272C>G | p.Thr91Ser | 2 | rs771527214 | Triebwasser et al. 2015 | N/D | 23.3 | probably damaging |
| 496C>T | p.Arg166Trp | 3 | NA | Triebwasser et al. 2015 | N/D | 25.2 | probably damaging |
| 695G>A | p.Arg232Gln | 4 | NA | Triebwasser et al. 2015 | N/D | 25.6 | possibly damaging |

Supplementary Table 5: A summary of the characterisation of the FH 1-4 variants compared to WT

| **Mutant** | **Cofactor Activity** | **Approximate C3b Binding Affinity (*K*_D_) (µM) in PBST-HBST** | **DAA (SPR)** | **TMC Formation (SPR)** |
| --- | --- | --- | --- | --- |
| WT | - | 10-10.1 | - | - |
| P26S | ns | 11.5-12 | ↓ | ↓ |
| R83S | ↓↓↓  P = 0.0006 | NC | ↓↓↓ | ↓↓↓ |
| T91S | ns | 9.4-11.7 | ↓ | ↓ |
| R166W | ↓↓  P = 0.0075 | 16.7-20.9 | ↓ | ↓↓ |
| R232Q | ↓↓↓  P = 0.0082 | NC | ↓ | ↓↓↓ |

ns= non significant; ND = not calculable.

Supplementary Table 6: A summary of the characterisation of the FI variants compared to WT

| **Mutant** | **Cofactor Activity**  **(FH cofactor)** | **Sheep cofactor**  **IC50 (nM)** | **TMC Formation (SPR)** |
| --- | --- | --- | --- |
| WT | - | 1.637nM | - |
| I340T | ↓↓↓ | ND | ↓↓↓ |
| H380R | ↓↓↓ | ND | ↓ |
| R406H | ns | 2.039nM  ns | ↓ |
| K441R | ns | 1.556nM  ns | ↓ |
| P553S | ↓  P = 0.045 | 1.664nM  ns | ↓↓ |

ns= non significant; ND = not done.
